# Deep-sea shipwrecks represent island-like ecosystems for marine microbiomes

**DOI:** 10.1038/s41396-021-00978-y

**Published:** 2021-04-22

**Authors:** Leila J. Hamdan, Justyna J. Hampel, Rachel D. Moseley, Rachel. L. Mugge, Anirban Ray, Jennifer L. Salerno, Melanie Damour

**Affiliations:** 1 University of Southern Mississippi, Ocean Springs, MS USA; 2 George Mason University, Manassas, VA USA; 3 Bureau of Ocean Energy Management, New Orleans, LA USA

**Keywords:** Biogeography, Microbial biooceanography

## Abstract

Biogeography of macro- and micro-organisms in the deep sea is, in part, shaped by naturally occurring heterogeneous habitat features of geological and biological origin such as seeps, vents, seamounts, whale and wood-falls. Artificial features including shipwrecks and energy infrastructure shape the biogeographic patterns of macro-organisms; how they influence microorganisms is unclear. Shipwrecks may function as islands of biodiversity for microbiomes, creating a patchwork of habitats with influence radiating out into the seabed. Here we show microbiome richness and diversity increase as a function of proximity to the historic deep-sea shipwreck *Anona* in the Gulf of Mexico. Diversity and richness extinction plots provide evidence of an island effect on microbiomes. A halo of core taxa on the seabed was observed up to 200 m away from the wreck indicative of the transition zone from shipwreck habitat to the surrounding environment. Transition zones around natural habitat features are often small in area compared to what was observed at *Anona* indicating shipwrecks may exert a large sphere of influence on seabed microbiomes. Historic shipwrecks are abundant, isolated habitats with global distribution, providing a means to explore contemporary processes shaping biogeography on the seafloor. This work is a case study for how built environments impact microbial biodiversity and provides new information on how arrival of material to the seafloor shapes benthic microbiomes.

## Introduction

Like all organisms, microorganisms exhibit biogeographic patterns (i.e., non-random distribution over space and time). In the last decade, strides have been made towards describing the biogeography of microorganisms in a variety of environments [1–7]. Although studies have examined microbial biogeography in coastal and marine habitats, the deep ocean is a frontier for hypothesis-driven experimental design, due to access limitation. In most habitats, historical events (evolution of microbiomes in isolated habitats) and the contemporary environment (local physical and geochemical features) are attributed with shaping microbial biogeographic patterns [5], although processes driving patterns are not clear. Pelagic dispersal and environmental selection (physical and chemical cues) are key to dictating microbial biogeographic patterns in the aquatic realm [3, 6, 8]. Different drivers of microbial biogeography are the subject of lively debate; however, the majority of debate focuses on natural features. Built features (i.e., structures created or modified by humans) have received significantly less study.

Hanson et al. [3] reviewed environmental microbial biogeography studies, finding the influence of physical and chemical factors on community composition and assembly to be significant. While features associated with hydrodynamics including density, salinity, temperature bathymetry and circulation [9] impact dispersal and biodiversity of all aquatic organisms, correlation between microbial composition and habitat features exists in 92% of studies reviewed [3]. This demonstrates habitat features shape microbial biogeographic patterns. In the deep sea, habitat features including methane seeps, vents and seamounts play a role in dictating microbial distribution and dispersal on the seabed [1, 10–13]. The built environment in the deep ocean, including historic shipwrecks (>50 years old), has not been studied for its influence on microbial biogeography.

Shipwrecks become artificial reefs and islands of biological diversity [14–17]. The biogeographic distribution of macro-organisms is shaped by the presence of shipwrecks as island-like systems on the seabed [18]. Island Theory of Biogeography [19] holds that in island-like systems (i.e., locations disconnected from similar environments) species richness and diversity are determined by the size of the ‘island’ and its isolation from a ‘mainland’ source of taxa or other ‘islands’. These features of isolation and size dictate species immigration and extinction rates. Multiple facets of Island Theory have been addressed in terrestrial habitats, lakes, and to a lesser degree, artificial reefs [20]. There is evidence that hard habitat features in the deep sea separated from other habitats by sediment can be considered island-like [13]. Shipwrecks, which are transformed into artificial reefs through colonization by microorganisms, who establish and preserve the habitability of the built structure, arguably function as islands in the deep sea, with their presence yielding a biodiversity spillover effect on the surrounding seafloor [21]. Study of built seafloor microbiomes offers opportunity to explore foundational communities in the deep sea and test ecological theories in novel ways. The study of shipwreck associated microbiomes, specifically, will yield knowledge on ecological value and health of artificial reefs on the seabed [22]. The goal of this study is to evaluate if shipwrecks function as island-like systems for benthic microbiomes. We hypothesize built structures, including shipwrecks, decrease microbiome isolation on the seabed. Here we show microbiome richness and diversity increase as a function of proximity to a historic shipwreck, and that the shipwreck leaves a halo of core taxa on the seabed, indicative of influence on the surrounding environment.

**Fig. 1:**
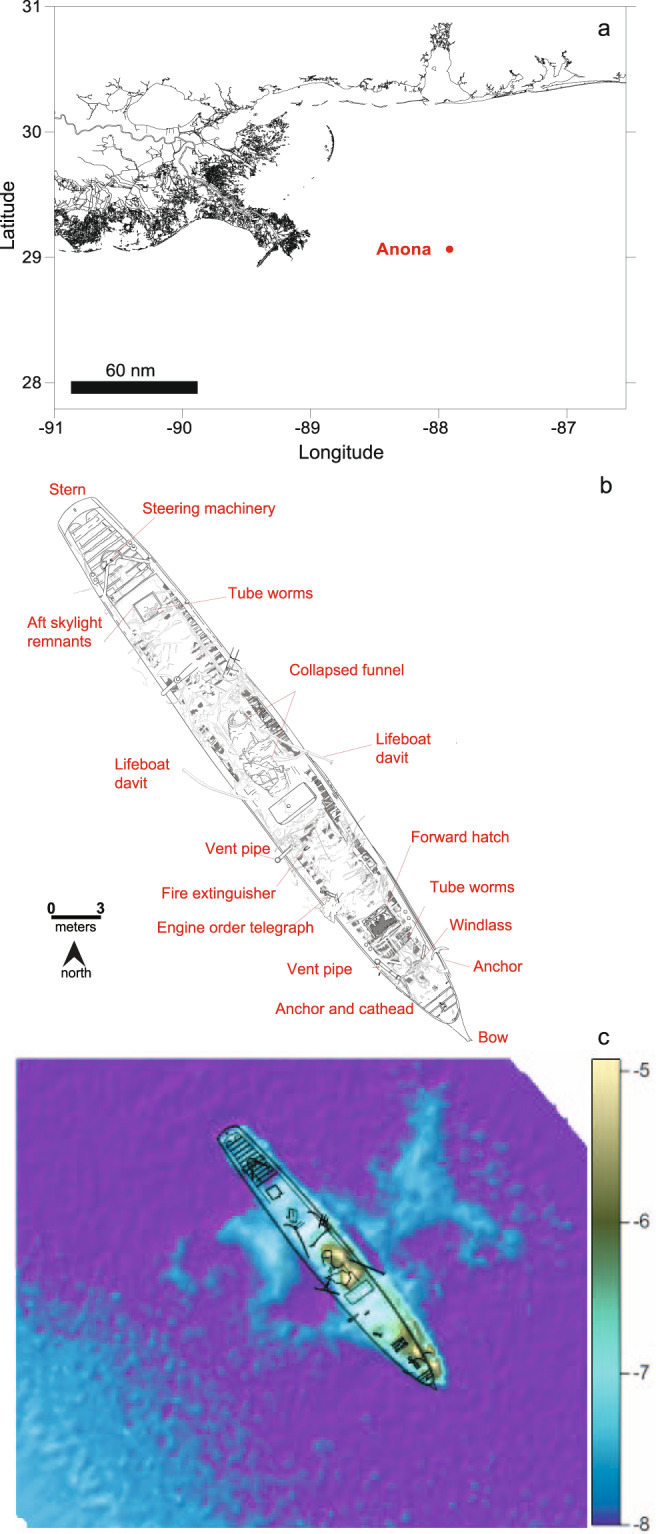
Study area in the northern Gulf of Mexico and site information. Location of historic shipwreck *Anona* (**a**). Archaeological site plan of *Anona* (**b**). Multibeam data collected in 2014 with a Kongsberg EM 2040 flown over the site using an autonomous underwater vehicle (AUV) at 1250 m (**c**). Shading on panel **c** shows difference in altitude off the seabed of the AUV. Geotagged site plan for *Anona* is draped over multibeam data to provide scale. Coordinates on panels **b** and **c** intentionally omitted to protect the exact location of the shipwreck.

This work is a case study focused on one extensively sampled shipwreck, *Anona* (Fig.1), resting at ~1258 m in the northern Gulf of Mexico. *Anona* was a twin masted, steel-hulled, luxury steam yacht that was built in 1904 and sank in 1944 [23]. The steam-propelled vessel carried no fuel—the only cargo was potatoes destined for the West Indies. The wreckage was discovered in 1995 and underwent a geophysical survey in 2002. *Anona* has been visually inspected via remotely operated vehicle (ROV) on several occasions, three during this study [23], and by multibeam echosounder mounted on an autonomous underwater vehicle (AUV), once during this study. The shipwreck measures 42 m long by 5 m wide with 2.2 m of vertical relief. It is oriented on the seabed with the bow facing southeast, upright and listing to starboard (Fig. 1). Multibeam data collected in 2014 with a Kongsberg EM 2040 shows a prominent sediment berm on the starboard side towards the stern, extending ~5 m from the shipwreck. This berm likely formed from sediment displaced when the hull impacted the seabed. There is wreck debris on the seabed in this area. There is also a less prominent sediment berm on the port side of the shipwreck extending ~15 m from the site. North of the wreck, thirteen sonar contacts were noted in 2002 and are likely debris dislodged as *Anona* sank on its southward trajectory. The study of this site sets the stage for investigating how built structures shape microbiomes in the deep marine biosphere.

## Methods

### Sample collection

Sediment was collected during seven expeditions, four on the Research Vessel (R/V) *Pelican* (PE14-15 [March 2014], PE15-02 [July 2014], PE15-22 [April 2015], PE16-23 [May 2016]) and three on R/V *Point Sur* (PS17-26 [June 2017], PS18-27 [June 2018], PS19-06 [September 2018]). Samples from PE15-22, PE16-23, PS17-26 and PS18-27 were collected using a MC800 deep-sea multi-corer (Ocean Instruments) fitted with a Tracklink Ultra-short baseline (USBL) transponder to provide positional information relative to *Anona*. Samples from PE14-15, PE15-02 and PS19-06 were collected using ROV *Global Explorer* (Oceaneering) and *Odysseus* (Pelagic Research Services) respectively. Prior to collection, visual surveys were performed to update the archaeological site plan (Fig. 1) and identify areas for sampling devoid of archaeological materials. SONAR on the ROV provided range to the shipwreck, and collection location was verified using USBL on the ROVs. The ROV’s 7-function manipulator arm was used to deploy Jason-style push cores. These efforts resulted in 23 cores collected at 2–1000 m from *Anona*, on four transects radiating northwest, southeast, northeast and southwest from the ship’s center. Cores were immediately sectioned at 2–4 cm intervals using an extruder, placed in conical vials and frozen at −80 °C.

Samples from two additional shipwrecks were collected during the same expeditions, using the methods described above. The details on sample date and approach are provided in Table S1. The use of samples from shipwrecks *Halo* and *Alcoa Puritan* in this study was restricted to their application in a machine learning exercise to identify amplicon sequence variants (ASVs) associated with proximity to large (>40 m long) steel-hulled shipwrecks with wrecking events dating to the early 1940s. The shipwrecks *Halo* and *Alcoa Puritan*, both sunk by German U-boat torpedo strikes in 1942, are described elsewhere [22, 23]. Samples were collected at distances spanning 2–200 m from the hulls of *Halo* and *Alcoa Puritan*. *Halo* had one complete east – west transect and *Alcoa Puritan* included three transects, clustered on one side of the wreckage to avoid artifacts and debris surrounding the site. As both lack a complete radial transect sampling design, similar to *Anona*, their application in this work is limited.

### Molecular analyses, bioinformatics and statistics

Samples were analyzed as previously described [22, 24]. Genomic DNA from sediment was extracted with the FastDNA SPIN kit (MP Biomedical Inc.), and used at the Integrated Microbiome Resource (IMR) facility at Dalhousie University (Halifax, Nova Scotia, Canada) for gene amplification and sequencing [25] using primers targeting the V6–V8 variable regions of the bacterial 16S rRNA gene. PCR was performed in duplicate, products were pooled, cleaned, and normalized with the Invitrogen SequalPrep 96-well Kit. Samples were quantified, and analyzed on an MiSeq platform (Illumina) generating 300 bp paired-end sequences. Bioinformatics were carried out using Quantitative Insights into Microbial Ecology (QIIME2) [26]. DADA2 was used for quality control and chimera removal and produced an ASV feature table [27] generated with quality based sequence truncation to remove low quality regions of sequences, but without a set trim length to preserve sequence information. The ASV table was used for taxonomic identification against the SILVA version 132 reference using VSEARCH and clustering at 99%.

Statistical analyses were carried out using PRIMER v. 6.1.13 (PRIMER-E Ltd., Plymouth, UK). Bray–Curtis dissimilarities were calculated from square-root transformed abundance data for ordination using non-metric multidimensional scaling (nMDS) to yield a “best fit” 2-dimensional representation of community structure. This representation was checked against a Principal Coordinates Analysis (PCoA) of Bray–Curtis data. Hierarchical clustering analysis (CLUSTER) generated similarity dendrograms and the analysis of similarity (ANOSIM) tested for differences between clusters. The similarity percent analysis (SIMPER) assisted with identifying phylotypes responsible for difference in clusters.

To test for island effects, diversity–, taxa– and evenness–extinction plots were created with alpha-diversity metrics derived from the ASV table. The core microbiome was resolved with the core features plugin in QIIME2. The q2-sample-classifier in QIIME2 made predictions of ASVs associated with proximity to *Halo* and *Alcoa Puritan*. The regress-samples function and the Random Forest Regressor estimator generated a feature importance table and model summary. The feature importance table was used to filter the *Anona* ASV table for ASVs identified as “important features” for metal-hulled shipwrecks. Surfer (Golden Software) was used to create areal diversity plots.

Sequences for *Anona* (2015-2018) and *Alcoa Puritan* are provided under NCBI Bioproject Number PRJNA612314. Sequences for *Halo*, and 2014 samples for *Anona* are found under NCBI Bioproject Number PRJNA401282.

## Results

A total of 138 sediment samples were collected around *Anona* generating over 4 million sequences prior to quality control and ~700,000 after. The average number of post quality control sequences per sample from *Anona* was ~5000. Samples from *Halo* and *Alcoa Puritan* were used to support machine learning predictions (Table S1). In total, 255 samples were incorporated in this study.

The focus of the work was to quantify how proximity to a shipwreck affects bacterial community diversity and composition. A Permutational multivariate analysis of variance (PERMANOVA) tested the hypothesis that microbiome structure is shaped by distance from *Anona* (Table 1). The influence of sediment depth was also addressed. Both distance and depth were significant structuring features on communities. The interaction between the two features was not significant.

**Table 1:** Permutational multivariate analysis of variance (PERMANOVA) conducted on sediment samples to determine differences in microbiome community similarity based on depth (cm below seafloor or cmbsf) and distance (m) from Anona. PERMANOVA was run using Type III (partial) sum of squares, fixed effects summed to zero with permutation of residuals under a reduced model and 999 permutations.

Source	Degrees of freedom	Sum of squares	Mean sum of squares	Pseudo-F	Significance	Permutations
Depth (cmbsf)	13	45,985	3535	3.24	0.00	997
Distance (m)	15	33,835	2255	2.06	0.00	997
Depth x Distance	91	93,994	1032	0.95	0.72	997
Residuals	18	19,677	1124			
Total	137	196,630				

**Fig. 2:**
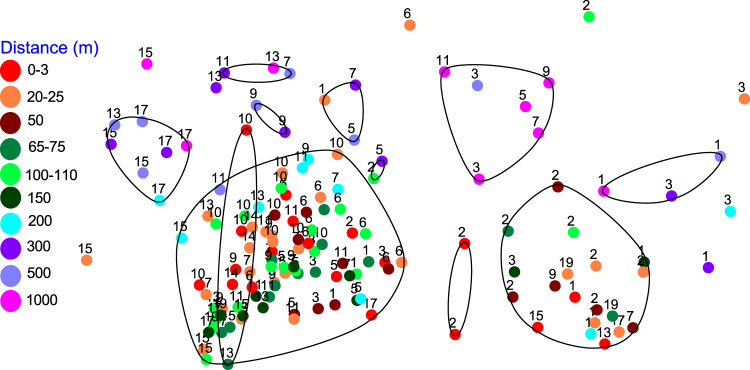
Non-metric multidimensional scaling analysis (nMDS) for bacterial 16S rRNA gene Bray–Curtis similarity data (after square root transformation) in samples collected around *Anona*. Samples were grouped in distance categories spanning up to 10 m for visualization. Numbers over symbols indicate average depth in cm below seafloor. CLUSTER analysis generated similarity contours at 50% (black lines). The 2D nMDS stress for this analysis is 0.13.

Samples were visualized by nMDS to determine ordination of samples based on depth and distance (Fig. 2). Based on the study design, samples were binned in groups spanning 0–10 m. Bacteria formed distinct groups based primarily on distance from the shipwreck. Samples collected 0–200 m from *Anona* formed four clusters. The largest cluster spanned the 0–200 m distance. The second largest contained samples collected within 75 m of the shipwreck. Most samples collected 300–1000 m away formed smaller groups organized loosely by sediment depth (Fig. 2). A PCoA on Bray–Curtis data had a highly similar ordination (not shown) and the first three axes explain ~6% the variation in the data with distance from the shipwreck and sediment depth featuring in the ordination. ANOSIM (Table S2) revealed samples collected 300 m or more from *Anona* were statistically different than all groups 200 m or less from *Anona*.

Up to 21% (average 17 ± 2%) of ASVs were classified only at the phylum level as Proteobacteria (Fig. S1). Gammaproteobacteria averaged 11% of the community at all distances (±3%), followed by Phycisphaerae and Deltaproteobacteria (both 10 ± 2% and Alphaproteobacteria (8 ± 1%). The similarity percent analysis (SIMPER) reveals an uncultured Proteobacteria phylotype accounts for between 24–40% of within distance group similarity in all sample groups (Table S3).

**Fig. 3:**
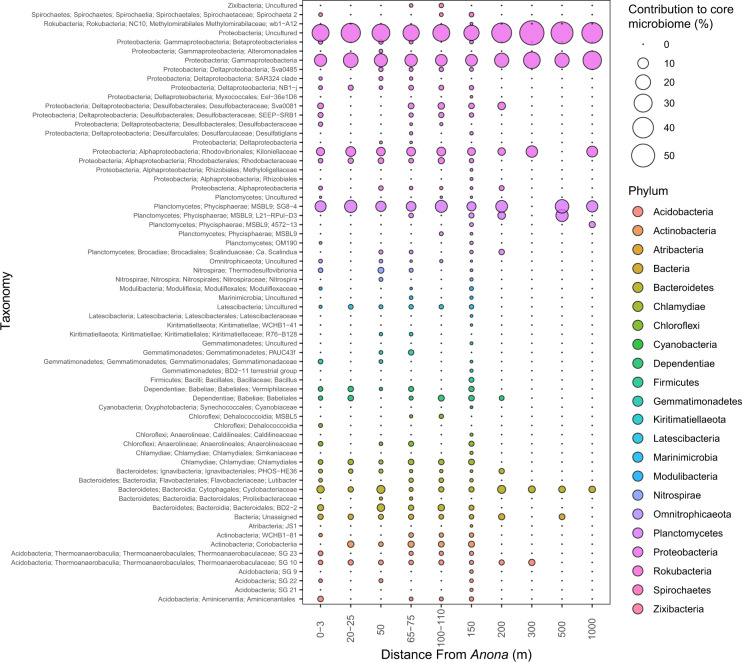
Bacterial core microbiome membership around Anona. Samples are grouped in distance categories spanning 10 m. Bubbles represent the relative contribution to the core community.

The core microbiome of each distance group was analyzed to understand the persistence of specific phylotypes around the shipwreck. Membership in the core microbiome was based on detection in 80% of samples in each group. The number of core members ranged from 7 to 62, with the fewest in samples at or greater than 300 m from *Anona* (Fig. 3). Conversely, core membership was most numerous at 150 m followed by 65–75 m and 0–3 m from the shipwreck. There were 15 unique core members only found at 150 m from the shipwreck. The Proteobacteria phylotype not annotated below phylum, along with a Gammaproteobacteria not annotated below class were significant contributors to the core microbiome at all distances.

**Fig. 4:**
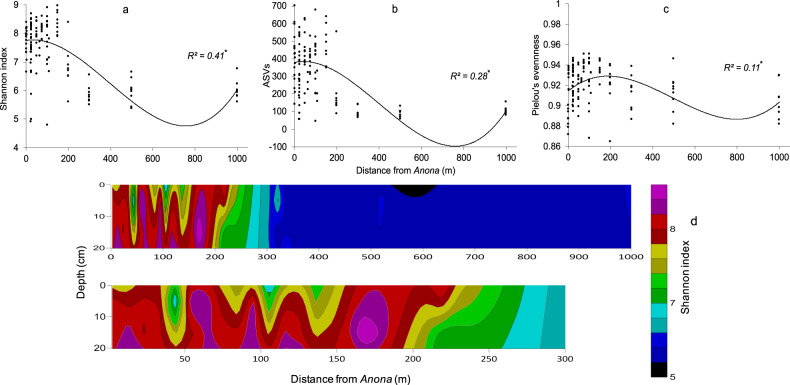
Shipwreck proximity and alpha diversity relationships in sediment. Diversity – isolation (**a**) richness – isolation (**b**) and evenness – isolation (**c**) curves. Significance (⍴ > 0.00) of polynomial regression indicated by *. Spatial analysis of bacterial diversity adjacent to *Anona* (**d**). Contours constructed from data from four transects aggregated on one axis. Grid created with the Kriging method applied to observed data.

Regression analysis was used to aid in understanding if microbiome richness and diversity increase as a function of proximity to *Anona*. The analysis included all samples from all sediment depths on all four transects around the site. Significant relationships between distance and bacterial richness, diversity, and evenness were observed (Fig. 4) although the trend for evenness was not strong. Average Shannon index (8.44), richness (532) and Pileou’s evenness (0.93) were highest 150 m from the shipwreck. The relationship between distance and Shannon index was stronger for deeper samples (9–19 cmbsf, *R*^2^ = 0.54 ρ > 0.00) than surface samples (0–8 cmbsf, *R*^2^ = 0.29 ρ > 0.00). A cross section of diversity interpolated from data from all four transects combined predicts diversity is highest in deeper sediments. At ~18–19 cm below the seafloor (cmbsf) at several locations within ~170 m from the site, diversity hotspots are predicted (Fig. 4). The sedimentation rate at *Anona*, determined by ^210^Pb analysis on cores from cruise PE15-02 is 0.26 cm per year [22]. A depth of 18–19 cmbsf is consistent with *Anona’s* year of arrival on the seafloor.

**Fig. 5:**
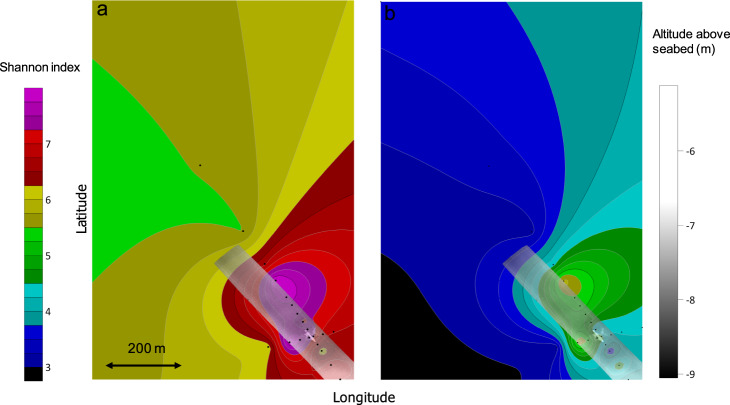
Areal Shannon index around the shipwreck *Anona*. Core sampling around the shipwreck *Anona* superimposed over (**a**) color contours depicting average whole core bacterial diversity and (**b**) diversity of ASVs predicted to be correlated with proximity to metal-hulled shipwrecks. Color contours were generated with the Kriging method applied to observations. Black circles identify where sediment samples were collected. The top layer (gray rectangle) is multibeam data. Shading depicts altitude of the AUV carrying the multibeam above the seabed when flown at a depth of 1250 m. Coordinates are intentionally omitted from the figure to protect the location of the shipwreck.

The areal plot of the Shannon index (Fig. 5) predicts diversity is elevated around the shipwreck, especially north and west of the site. A sediment berm associated with the wrecking event is located west of the site (Fig. 1), potentially evidencing transport of sediment microbiomes into the surrounding seabed [23]. The area northwest of the shipwreck contains sonar targets, potentially pointing to influences of smaller debris fields dislodged during the wrecking event on microbiomes. The ASV table for *Anona* was filtered for features predicted to be associated with proximity to other metal-hulled shipwrecks (*Halo* and *Alcoa Puritan*) [22, 23]. The model summary for the prediction is provided in Table S4. A halo of biodiversity for taxa correlated with metal-hulled shipwrecks is apparent 25–200 m away from the site, also focused north and west of the shipwreck.

## Discussion

This study is the first to provide a molecular view of bacterial richness and diversity in the seabed surrounding a built structure in the deep marine biosphere. Around *Anona*, diversity and richness increase with proximity to the shipwreck in all directions providing evidence of an island effect on marine benthic microbiomes from a built feature. *Anona’s* site formation event occurred 76 years ago, and the sampling approach used does not permit a complete understanding of the rate at which shipwreck island effects on the bacterial microbiome emerged. However, the “halo” of elevated diversity of both the whole community and core community at intermediate distances along the transect provide clues to how time and physical disturbances impact sediment microbiomes. The co-occurrence of high diversity at depths consistent with the site formation event, where berms formed when the shipwreck impacted the seafloor and where debris dislodged during the wrecking event, reveal the initial encounter has left a persistent signature of disturbance for nearly 8 decades.

Stieglitz [21] observed biological “halos” around shallow shipwrecks, indicated by distinct benthic macrobiological communities relative to the surrounding seafloor, and elevated biodiversity of benthic invertebrates. The size of the “halos” ostensibly, how far they moved away from shipwrecks into the surrounding seafloor, was a function of time since the site’s original formation. Stieglitz explained the “halos” increased in size with time, possibly in response to resource depletion during hull degradation. They presented another scenario, that dispersal of organisms from the shipwreck resulted in the macrobiological halo. Both of these ideas may explain the observed elevated biodiversity at a standoff distance from *Anona*. In the current study, the microbiome may follow macrobenthic distribution in sediment, although that was not explicitly addressed in this work. It is also possible that the microbiome associated with the wreck disperses over time, forming a colonization front that eventually converges with the “background” seafloor microbiome [6, 28]. Such a convergence aligns with the concept of an “ecotone” or transition zone connecting adjacent ecosystems along temporal and spatial gradients ([29] and references therein). Microbial ecotones have been observed in deep sea sediments surrounding a variety of habitat features.

Geological features on the seabed, including vents and seeps are numerous, and often spatially isolated from each other. Vents, seeps and whale falls impact local biogeochemistry in different ways but all present a physical habitat unlike the surrounding seafloor and provide complex substrates for fauna to colonize. Whale falls have higher microbiome diversity proximate to the fall relative to non-fall reference sites [30, 31]. Whale falls also show increased seabed biodiversity with a transition zone from fall habitat to background habitat occurring within 10 m of a 15-year old fall [32]. The distance an ecotone presents away from methane seeps featuring chemosynthetic benthic assemblages depends on the size of the seep and gas/fluid flux, and can occur up to 100 m away [29, 33]. While the shipwreck under study in this work is historic (>50 years old) the temporal scale of its presence on the seafloor is more aligned with ephemeral biological falls than vents and seeps which form on geological time scales. Nevertheless, the size of the transition zone around *Anona* is larger than reported for some seeps [33] where analogous sampling strategies were used. This may indicate that the dispersal sphere of influence of shipwrecks on the seabed is large even with the decadal time scales of such sites. The size and relief of shipwrecks above the seafloor may also create greater encounter opportunities for both benthic and pelagic taxa [13].

The distance the shipwreck microbiome extends away from the site may also be influenced by active dispersal of sediment during site formation. Active dispersal may explain observations of high diversity to the west of the shipwreck where a sediment berm formed during the wreckage event is evident (Fig. 1), and debris is present on the seabed. In addition, depending on size, the presence of a shipwreck can impact local hydrodynamics, resulting in deposition of sediment, organic matter, creation of areas of protection from disturbances, or development of soft sediment berms [34] that are more easily colonized. Evidence of this is provided by multibeam data and the more shallow sediment berm to the east of the site (Fig. 1).

The investigation of the benthic microbiome around the shipwreck *Anona* is a case study for understanding how the built environment impacts microbial biodiversity on the seabed. The data we present indicate the sphere influence of the shipwreck on the benthic microbiome extends 200 m into the surrounding environment. This is an interesting finding in isolation; however, *Anona* does not exist as a singular entity. In the Gulf of Mexico alone, there are more than 2000 known historic shipwrecks, the earliest having come to rest on the seabed 500 years ago [35]. A majority of these known shipwrecks date to the 19th century presenting more than 100 years of influence on the seabed. These are joined with >7100 oil and gas-related structures installed since 1942, and ~45,000 miles of oil and gas pipeline [36, 37]. While the cultural, historical, and archaeological value of shipwrecks is established, their role and that of other built structures in marine ecosystem processes is only beginning to emerge.

Indeed, shipwrecks function as artificial reefs and hotspots of biological diversity; their effect on microbiome diversity however remains unclear. Species richness and endemicity—key metrics in the study of biogeography—are controlled in part by habitat complexity. Learning how habitat density of built features will impact microbiome diversity on the seabed is of importance. In areas with patchy habitat, distinct microbiomes are separated by unsuitable areas that must be traversed [38]. On the seabed, microorganisms employ passive dispersal to move between habitats, but the probability of arrival at a like habitat is controlled by isolation. With increased habitat diversity, i.e., more built features [14] spatial isolation decreases, possibly resulting in greater microbiome diversity on small scales. Alternately, past a certain threshold, the addition of built habitats to the seabed could result in high habitat density, homogeneity and overlapping transition zones, thus reducing diversity. This study highlights the need to address these concepts, given the large footprint of just one shipwreck on the seafloor microbiome.

## Supplementary information

Supplementary Figure and Tables

Figure S1
