# Supplementary material for: Deep-sea shipwrecks represent island-like ecosystems for marine microbiomes
Source: ISME J. 2021 Apr 22;15(10):2883–91. doi: 10.1038/s41396-021-00978-y (PMC8443566; doi:10.1038/s41396-021-00978-y)
Supplement: Supplementary file 1 — Supplementary Figure and Tables [file 41396_2021_978_MOESM1_ESM.pdf]

Deep-sea shipwrecks represent island-like ecosystems for marine microbiomes

Leila J. Hamdan[1*], Justyna J. Hampel[1], Rachel D. Moseley[1], Rachel. L Mugge[1], Anirban Ray[1], Jennifer L. Salerno[2], Melanie Damour[3]

[1]University of Southern Mississippi, Ocean Springs, MS 39564

[2]George Mason University, Manassas, VA 22030

[3] Bureau of Ocean Energy Management, New Orleans, LA, 70123

*Corresponding author: Leila J. Hamdan, University of Southern Mississippi, School of Ocean Science and Engineering, Ocean Springs, MS, USA. e-mail: leila.hamdan@usm.edu. Tel: (228) 818-8011

Supplementary Information

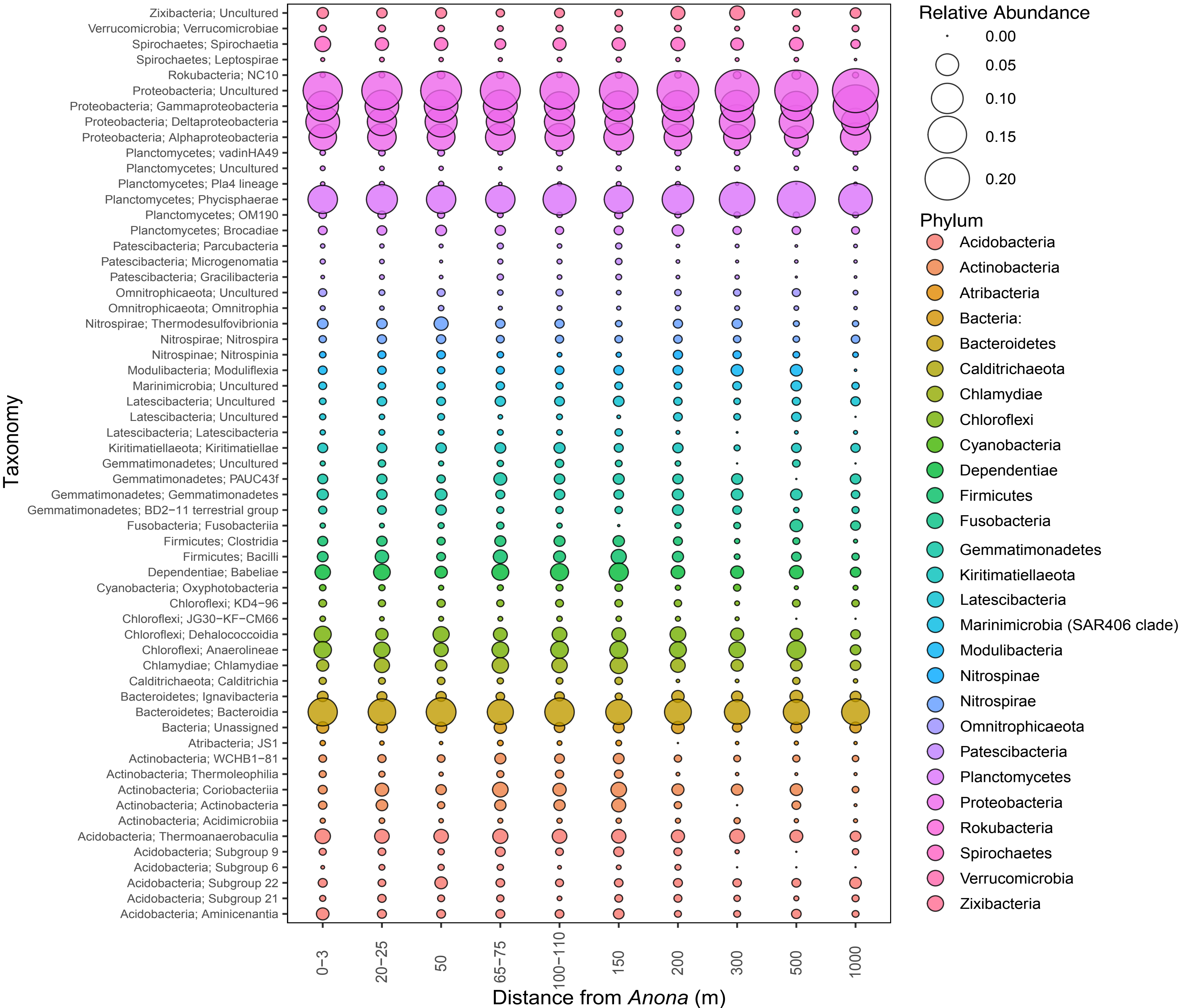

Fig. S1. Class level (L3) composition of bacteria around Anona. Samples are grouped in distance categories spanning 10 m. Classes accounting for less than 1% of the total microbiome were omitted for ease of visualization.

Table S1. Detailed information on samples included in study.

Sample ID	Site	Depth	Distance	Transect	Direction	HullType	Sample Da	Sampling Approach
Anona-200	Anona	1	2	4	NW	Metal	3/20/14	ROV - Push Core
Anona-201	Anona	3	2	4	NW	Metal	3/20/14	ROV - Push Core
Anona-202	Anona	5	2	4	NW	Metal	3/20/14	ROV - Push Core
Anona-203	Anona	7	2	4	NW	Metal	3/20/14	ROV - Push Core
Anona-204	Anona	9	2	4	NW	Metal	3/20/14	ROV - Push Core
Anona-205	Anona	11	2	4	NW	Metal	3/20/14	ROV - Push Core
Anona-36	Anona	13	2	4	NW	Metal	3/20/14	ROV - Push Core
Anona-37	Anona	15	2	4	NW	Metal	3/20/14	ROV - Push Core
Anona-38	Anona	17	2	4	NW	Metal	3/20/14	ROV - Push Core
Anona-39	Anona	1	50	4	NW	Metal	4/29/15	Multicore
Anona-40	Anona	3	50	4	NW	Metal	4/29/15	Multicore
Anona-41	Anona	5	50	4	NW	Metal	4/29/15	Multicore
Anona-412	Anona	7	50	4	NW	Metal	4/29/15	Multicore
Anona-413	Anona	9	50	4	NW	Metal	4/29/15	Multicore
Anona-414	Anona	11	50	4	NW	Metal	4/29/15	Multicore
Anona-415	Anona	1	25	4	NW	Metal	5/18/16	Multicore
Anona-416	Anona	3	25	4	NW	Metal	5/18/16	Multicore
Anona-417	Anona	5	25	4	NW	Metal	5/18/16	Multicore
Anona-418	Anona	7	25	4	NW	Metal	5/18/16	Multicore
Anona-419	Anona	9	25	4	NW	Metal	5/18/16	Multicore
Anona-42	Anona	11	25	4	NW	Metal	5/18/16	Multicore
Anona-420	Anona	13	25	4	NW	Metal	5/18/16	Multicore
Anona-421	Anona	15	25	4	NW	Metal	5/18/16	Multicore
Anona-422	Anona	17	25	4	NW	Metal	5/18/16	Multicore
Anona-423	Anona	19	25	4	NW	Metal	5/18/16	Multicore
Anona-424	Anona	1	75	4	NW	Metal	5/18/16	Multicore
Anona-425	Anona	3	75	4	NW	Metal	5/18/16	Multicore
Anona-426	Anona	5	75	4	NW	Metal	5/18/16	Multicore
Anona-427	Anona	7	75	4	NW	Metal	5/18/16	Multicore
Anona-428	Anona	9	75	4	NW	Metal	5/18/16	Multicore
Anona-429	Anona	11	75	4	NW	Metal	5/18/16	Multicore
Anona-43	Anona	13	75	4	NW	Metal	5/18/16	Multicore
Anona-430	Anona	15	75	4	NW	Metal	5/18/16	Multicore
Anona-431	Anona	17	75	4	NW	Metal	5/18/16	Multicore
Anona-432	Anona	19	75	4	NW	Metal	5/18/16	Multicore
Anona-433	Anona	1	100	4	NW	Metal	5/18/16	Multicore
Anona-434	Anona	3	100	4	NW	Metal	5/18/16	Multicore
Anona-435	Anona	5	100	4	NW	Metal	5/18/16	Multicore
Anona-436	Anona	7	100	4	NW	Metal	5/18/16	Multicore
Anona-437	Anona	9	100	4	NW	Metal	5/18/16	Multicore
Anona-438	Anona	11	100	4	NW	Metal	5/18/16	Multicore
Anona-439	Anona	13	100	4	NW	Metal	5/18/16	Multicore
Anona-44	Anona	15	100	4	NW	Metal	5/18/16	Multicore
Anona-440	Anona	17	100	4	NW	Metal	5/18/16	Multicore
Anona-441	Anona	19	100	4	NW	Metal	5/18/16	Multicore
Anona-442	Anona	1	150	4	NW	Metal	5/18/16	Multicore
Anona-443	Anona	3	150	4	NW	Metal	5/18/16	Multicore
Anona-444	Anona	5	150	4	NW	Metal	5/18/16	Multicore
Anona-445	Anona	7	150	4	NW	Metal	5/18/16	Multicore
Anona-446	Anona	9	150	4	NW	Metal	5/18/16	Multicore
Anona-447	Anona	11	150	4	NW	Metal	5/18/16	Multicore
Anona-448	Anona	13	150	4	NW	Metal	5/18/16	Multicore
Anona-449	Anona	15	150	4	NW	Metal	5/18/16	Multicore
Anona-450	Anona	17	150	4	NW	Metal	5/18/16	Multicore
Anona-451	Anona	19	150	4	NW	Metal	5/18/16	Multicore

Anona-730	Anona	1	25	4	NW	Metal	6/15/17	Multicore
Anona-731	Anona	3	25	4	NW	Metal	6/15/17	Multicore
Anona-733	Anona	7	25	4	NW	Metal	6/15/17	Multicore
Anona-734	Anona	9	25	4	NW	Metal	6/15/17	Multicore
Anona-735	Anona	11	25	4	NW	Metal	6/15/17	Multicore
Anona-736	Anona	13	25	4	NW	Metal	6/15/17	Multicore
Anona-737	Anona	15	25	4	NW	Metal	6/15/17	Multicore
Anona-738	Anona	17	25	4	NW	Metal	6/15/17	Multicore
Anona-740	Anona	1	200	4	NW	Metal	6/15/17	Multicore
Anona-741	Anona	3	200	4	NW	Metal	6/15/17	Multicore
Anona-742	Anona	5	200	4	NW	Metal	6/15/17	Multicore
Anona-743	Anona	7	200	4	NW	Metal	6/15/17	Multicore
Anona-744	Anona	9	200	4	NW	Metal	6/15/17	Multicore
Anona-745	Anona	11	200	4	NW	Metal	6/15/17	Multicore
Anona-746	Anona	13	200	4	NW	Metal	6/15/17	Multicore
Anona-747	Anona	15	200	4	NW	Metal	6/15/17	Multicore
Anona-748	Anona	17	200	4	NW	Metal	6/15/17	Multicore
Anona-950	Anona	1	300	4	NW	Metal	6/14/18	Multicore
Anona-951	Anona	3	300	4	NW	Metal	6/14/18	Multicore
Anona-952	Anona	5	300	4	NW	Metal	6/14/18	Multicore
Anona-953	Anona	7	300	4	NW	Metal	6/14/18	Multicore
Anona-954	Anona	9	300	4	NW	Metal	6/14/18	Multicore
Anona-955	Anona	11	300	4	NW	Metal	6/14/18	Multicore
Anona-956	Anona	13	300	4	NW	Metal	6/14/18	Multicore
Anona-957	Anona	15	300	4	NW	Metal	6/14/18	Multicore
Anona-958	Anona	17	300	4	NW	Metal	6/14/18	Multicore
Anona-960	Anona	1	500	4	NW	Metal	6/14/18	Multicore
Anona-961	Anona	3	500	4	NW	Metal	6/14/18	Multicore
Anona-962	Anona	5	500	4	NW	Metal	6/14/18	Multicore
Anona-963	Anona	7	500	4	NW	Metal	6/14/18	Multicore
Anona-964	Anona	9	500	4	NW	Metal	6/14/18	Multicore
Anona-965	Anona	11	500	4	NW	Metal	6/14/18	Multicore
Anona-966	Anona	13	500	4	NW	Metal	6/14/18	Multicore
Anona-967	Anona	15	500	4	NW	Metal	6/14/18	Multicore
Anona-968	Anona	17	500	4	NW	Metal	6/14/18	Multicore
Anona-970	Anona	1	1000	4	NW	Metal	6/14/18	Multicore
Anona-971	Anona	3	1000	4	NW	Metal	6/14/18	Multicore
Anona-972	Anona	5	1000	4	NW	Metal	6/14/18	Multicore
Anona-973	Anona	7	1000	4	NW	Metal	6/14/18	Multicore
Anona-974	Anona	9	1000	4	NW	Metal	6/14/18	Multicore
Anona-975	Anona	11	1000	4	NW	Metal	6/14/18	Multicore
Anona-976	Anona	13	1000	4	NW	Metal	6/14/18	Multicore
Anona-977	Anona	15	1000	4	NW	Metal	6/14/18	Multicore
Anona-978	Anona	17	1000	4	NW	Metal	6/14/18	Multicore
D-105	Anona	2	2.4	1	SE	Metal	9/11/18	ROV - Push Core
D-106	Anona	6	2.4	1	SE	Metal	9/11/18	ROV - Push Core
D-107	Anona	10	2.4	1	SE	Metal	9/11/18	ROV - Push Core
D-109	Anona	2	22	1	SE	Metal	9/11/18	ROV - Push Core
D-110	Anona	6	22	1	SE	Metal	9/11/18	ROV - Push Core
D-111	Anona	10	22	1	SE	Metal	9/11/18	ROV - Push Core
D-113	Anona	2	65	1	SE	Metal	9/11/18	ROV - Push Core
D-114	Anona	6	65	1	SE	Metal	9/11/18	ROV - Push Core
D-115	Anona	10	65	1	SE	Metal	9/11/18	ROV - Push Core
D-117	Anona	2	109	1	SE	Metal	9/11/18	ROV - Push Core
D-118	Anona	6	109	1	SE	Metal	9/11/18	ROV - Push Core
D-119	Anona	10	109	1	SE	Metal	9/11/18	ROV - Push Core
D-121	Anona	2	100	2	NE	Metal	9/11/18	ROV - Push Core
D-122	Anona	6	100	2	NE	Metal	9/11/18	ROV - Push Core

D-124	Anona	10	100	2	NE	Metal	9/11/18	ROV - Push Core
D-125	Anona	2	50	2	NE	Metal	9/11/18	ROV - Push Core
D-126	Anona	6	50	2	NE	Metal	9/11/18	ROV - Push Core
D-127	Anona	10	50	2	NE	Metal	9/11/18	ROV - Push Core
D-129	Anona	2	25	2	NE	Metal	9/11/18	ROV - Push Core
D-130	Anona	6	25	2	NE	Metal	9/11/18	ROV - Push Core
D-131	Anona	10	25	2	NE	Metal	9/11/18	ROV - Push Core
D-132	Anona	14	25	2	NE	Metal	9/11/18	ROV - Push Core
D-133	Anona	2	2.4	2	NE	Metal	9/11/18	ROV - Push Core
D-134	Anona	6	2.4	2	NE	Metal	9/11/18	ROV - Push Core
D-135	Anona	10	2.4	2	NE	Metal	9/11/18	ROV - Push Core
D-137	Anona	2	101	3	SW	Metal	9/11/18	ROV - Push Core
D-138	Anona	6	101	3	SW	Metal	9/11/18	ROV - Push Core
D-139	Anona	10	101	3	SW	Metal	9/11/18	ROV - Push Core
D-141	Anona	2	50	3	SW	Metal	9/11/18	ROV - Push Core
D-142	Anona	6	50	3	SW	Metal	9/11/18	ROV - Push Core
D-143	Anona	10	50	3	SW	Metal	9/11/18	ROV - Push Core
D-145	Anona	2	25	3	SW	Metal	9/11/18	ROV - Push Core
D-146	Anona	6	25	3	SW	Metal	9/11/18	ROV - Push Core
D-147	Anona	10	25	3	SW	Metal	9/11/18	ROV - Push Core
D-148	Anona	14	25	3	SW	Metal	9/11/18	ROV - Push Core
D-149	Anona	2	3.3	3	SW	Metal	9/11/18	ROV - Push Core
D-150	Anona	6	3.3	3	SW	Metal	9/11/18	ROV - Push Core
D-151	Anona	10	3.3	3	SW	Metal	9/11/18	ROV - Push Core
D-152	Anona	14	3.3	3	SW	Metal	9/11/18	ROV - Push Core
Halo-1	Halo	0	2	1	E	Metal	3/15/14	ROV - Push Core
Halo-166	Halo	0	2	1	E	Metal	7/24/14	ROV - Push Core
Halo-167	Halo	2	2	1	E	Metal	7/24/14	ROV - Push Core
Halo-168	Halo	4	2	1	E	Metal	7/24/14	ROV - Push Core
Halo-169	Halo	6	2	1	E	Metal	7/24/14	ROV - Push Core
Halo-170	Halo	8	2	1	E	Metal	7/24/14	ROV - Push Core
Halo-171	Halo	10	2	1	E	Metal	7/24/14	ROV - Push Core
Halo-172	Halo	12	2	1	E	Metal	7/24/14	ROV - Push Core
Halo-173	Halo	14	2	1	E	Metal	7/24/14	ROV - Push Core
Halo-174	Halo	16	2	1	E	Metal	7/24/14	ROV - Push Core
Halo-176	Halo	0	200	1	E	Metal	7/24/14	ROV - Push Core
Halo-177	Halo	2	200	1	E	Metal	7/24/14	ROV - Push Core
Halo-178	Halo	4	200	1	E	Metal	7/24/14	ROV - Push Core
Halo-179	Halo	6	200	1	E	Metal	7/24/14	ROV - Push Core
Halo-180	Halo	8	200	1	E	Metal	7/24/14	ROV - Push Core
Halo-181	Halo	10	200	1	E	Metal	7/24/14	ROV - Push Core
Halo-182	Halo	12	200	1	E	Metal	7/24/14	ROV - Push Core
Halo-183	Halo	14	200	1	E	Metal	7/24/14	ROV - Push Core
Halo-184	Halo	16	200	1	E	Metal	7/24/14	ROV - Push Core
Halo-2	Halo	2	2	1	E	Metal	3/15/14	ROV - Push Core
Halo-273	Halo	0	75	1	E	Metal	5/1/15	Multicore
Halo-274	Halo	2	75	1	E	Metal	5/1/15	Multicore
Halo-275	Halo	4	75	1	E	Metal	5/1/15	Multicore
Halo-276	Halo	6	75	1	E	Metal	5/1/15	Multicore
Halo-277	Halo	8	75	1	E	Metal	5/1/15	Multicore
Halo-278	Halo	10	75	1	E	Metal	5/1/15	Multicore
Halo-279	Halo	12	75	1	E	Metal	5/1/15	Multicore
Halo-280	Halo	14	75	1	E	Metal	5/1/15	Multicore
Halo-281	Halo	16	75	1	E	Metal	5/1/15	Multicore
Halo-286	Halo	0	150	1	E	Metal	5/1/15	Multicore
Halo-287	Halo	2	150	1	E	Metal	5/1/15	Multicore
Halo-288	Halo	4	150	1	E	Metal	5/1/15	Multicore
Halo-289	Halo	6	150	1	E	Metal	5/1/15	Multicore

Halo-290	Halo	8	150	1	E	Metal	5/1/15	Multicore
Halo-291	Halo	10	150	1	E	Metal	5/1/15	Multicore
Halo-292	Halo	12	150	1	E	Metal	5/1/15	Multicore
Halo-293	Halo	14	150	1	E	Metal	5/1/15	Multicore
Halo-3	Halo	4	2	1	E	Metal	3/15/14	ROV - Push Core
Halo-4	Halo	6	2	1	E	Metal	3/15/14	ROV - Push Core
Halo-482	Halo	0	50	1	E	Metal	5/20/16	Multicore
Halo-483	Halo	2	50	1	E	Metal	5/20/16	Multicore
Halo-484	Halo	4	50	1	E	Metal	5/20/16	Multicore
Halo-485	Halo	6	50	1	E	Metal	5/20/16	Multicore
Halo-486	Halo	8	50	1	E	Metal	5/20/16	Multicore
Halo-487	Halo	10	50	1	E	Metal	5/20/16	Multicore
Halo-488	Halo	12	50	1	E	Metal	5/20/16	Multicore
Halo-489	Halo	14	50	1	E	Metal	5/20/16	Multicore
Halo-490	Halo	16	50	1	E	Metal	5/20/16	Multicore
Halo-492	Halo	0	100	1	E	Metal	5/21/16	Multicore
Halo-493	Halo	2	100	1	E	Metal	5/21/16	Multicore
Halo-494	Halo	4	100	1	E	Metal	5/21/16	Multicore
Halo-495	Halo	6	100	1	E	Metal	5/21/16	Multicore
Halo-496	Halo	8	100	1	E	Metal	5/21/16	Multicore
Halo-497	Halo	10	100	1	E	Metal	5/21/16	Multicore
Halo-498	Halo	12	100	1	E	Metal	5/21/16	Multicore
Halo-499	Halo	14	100	1	E	Metal	5/21/16	Multicore
Halo-5	Halo	8	2	1	E	Metal	3/15/14	ROV - Push Core
Halo-500	Halo	16	100	1	E	Metal	5/21/16	Multicore
Halo-6	Halo	10	2	1	E	Metal	3/15/14	ROV - Push Core
Halo-7	Halo	12	2	1	E	Metal	3/15/14	ROV - Push Core
Halo-8	Halo	14	2	1	E	Metal	3/15/14	ROV - Push Core
Halo-9	Halo	16	2	1	E	Metal	3/15/14	ROV - Push Core
D153	Alcoa Puritan	4	100	1	WSW	Metal	9/13/18	ROV - Push Core
D154	Alcoa Puritan	8	100	1	WSW	Metal	9/13/18	ROV - Push Core
D155	Alcoa Puritan	12	100	1	WSW	Metal	9/13/18	ROV - Push Core
D156	Alcoa Puritan	16	100	1	WSW	Metal	9/13/18	ROV - Push Core
D157	Alcoa Puritan	4	50	1	WSW	Metal	9/13/18	ROV - Push Core
D158	Alcoa Puritan	8	50	1	WSW	Metal	9/13/18	ROV - Push Core
D159	Alcoa Puritan	12	50	1	WSW	Metal	9/13/18	ROV - Push Core
D161	Alcoa Puritan	4	25	1	WSW	Metal	9/13/18	ROV - Push Core
D162	Alcoa Puritan	8	25	1	WSW	Metal	9/13/18	ROV - Push Core
D163	Alcoa Puritan	12	25	1	WSW	Metal	9/13/18	ROV - Push Core
D164	Alcoa Puritan	16	25	1	WSW	Metal	9/13/18	ROV - Push Core
D165	Alcoa Puritan	4	6	1	WSW	Metal	9/13/18	ROV - Push Core
D166	Alcoa Puritan	8	6	1	WSW	Metal	9/13/18	ROV - Push Core
D167	Alcoa Puritan	12	6	1	WSW	Metal	9/13/18	ROV - Push Core
D168	Alcoa Puritan	16	6	1	WSW	Metal	9/13/18	ROV - Push Core
D169	Alcoa Puritan	4	3	2	W	Metal	9/13/18	ROV - Push Core
D170	Alcoa Puritan	8	3	2	W	Metal	9/13/18	ROV - Push Core
D171	Alcoa Puritan	12	3	2	W	Metal	9/13/18	ROV - Push Core
D173	Alcoa Puritan	4	3	2	W	Metal	9/13/18	ROV - Push Core
D174	Alcoa Puritan	8	3	2	W	Metal	9/13/18	ROV - Push Core
D175	Alcoa Puritan	12	3	2	W	Metal	9/13/18	ROV - Push Core
D176	Alcoa Puritan	16	3	2	W	Metal	9/13/18	ROV - Push Core
D177	Alcoa Puritan	4	25	2	W	Metal	9/13/18	ROV - Push Core
D178	Alcoa Puritan	8	25	2	W	Metal	9/13/18	ROV - Push Core
D179	Alcoa Puritan	12	25	2	W	Metal	9/13/18	ROV - Push Core
D180	Alcoa Puritan	16	25	2	W	Metal	9/13/18	ROV - Push Core
D181	Alcoa Puritan	4	50	2	W	Metal	9/13/18	ROV - Push Core
D182	Alcoa Puritan	8	50	2	W	Metal	9/13/18	ROV - Push Core
D183	Alcoa Puritan	12	50	2	W	Metal	9/13/18	ROV - Push Core

D184	Alcoa Puritan	16	50	2	W	Metal	9/13/18	ROV - Push Core
D185	Alcoa Puritan	4	100	2	W	Metal	9/13/18	ROV - Push Core
D186	Alcoa Puritan	8	100	2	W	Metal	9/13/18	ROV - Push Core
D187	Alcoa Puritan	12	100	2	W	Metal	9/13/18	ROV - Push Core
D188	Alcoa Puritan	16	100	2	W	Metal	9/13/18	ROV - Push Core
D189	Alcoa Puritan	4	2.5	3	SW	Metal	9/14/18	ROV - Push Core
D190	Alcoa Puritan	8	2.5	3	SW	Metal	9/14/18	ROV - Push Core
D191	Alcoa Puritan	12	2.5	3	SW	Metal	9/14/18	ROV - Push Core
D192	Alcoa Puritan	16	2.5	3	SW	Metal	9/14/18	ROV - Push Core
D193	Alcoa Puritan	4	25	3	SW	Metal	9/14/18	ROV - Push Core
D194	Alcoa Puritan	8	25	3	SW	Metal	9/14/18	ROV - Push Core
D195	Alcoa Puritan	12	25	3	SW	Metal	9/14/18	ROV - Push Core
D197	Alcoa Puritan	4	54.8	3	SW	Metal	9/14/18	ROV - Push Core
D198	Alcoa Puritan	8	54.8	3	SW	Metal	9/14/18	ROV - Push Core
D199	Alcoa Puritan	12	54.8	3	SW	Metal	9/14/18	ROV - Push Core
D200	Alcoa Puritan	16	54.8	3	SW	Metal	9/14/18	ROV - Push Core
D201	Alcoa Puritan	4	100	3	SW	Metal	9/14/18	ROV - Push Core
D202	Alcoa Puritan	8	100	3	SW	Metal	9/14/18	ROV - Push Core
D203	Alcoa Puritan	12	100	3	SW	Metal	9/14/18	ROV - Push Core
D204	Alcoa Puritan	16	100	3	SW	Metal	9/14/18	ROV - Push Core
D205	Alcoa Puritan	4	100	3	SW	Metal	9/14/18	ROV - Push Core
D206	Alcoa Puritan	8	100	3	SW	Metal	9/14/18	ROV - Push Core
D207	Alcoa Puritan	12	100	3	SW	Metal	9/14/18	ROV - Push Core
D208	Alcoa Puritan	16	100	3	SW	Metal	9/14/18	ROV - Push Core

Table S2. Analysis of similarity. Yellow highlighted boxes denote significant differences in communities between groups.

	0-3	50	20-25	65-75	100-110	150	200	300	500	1000
0-3										
50	0.01									
20-25	0.03	0.00								
65-75	0.80	0.13	-0.08							
100-110	0.07	0.14	-0.04	-0.01						
150	0.11	0.13	-0.07	-0.03	0.00					
200	0.07	0.07	0.80	0.19	0.16	0.10				
300	0.31	0.24	0.20	0.37	0.40	0.28	-0.03			
500	0.34	0.32	0.16	0.40	0.39	0.31	0.00	-0.67		
1000	0.33	0.19	0.18	0.45	0.47	0.36	0.11	0.40	0.14	

Table S3. Results of the Similarity Percentages Analysis

Group 0-3
Average similarity: 50.90

Species	Av.Abund	Av.Sim	Sim/SD	Contrib%	Cum.%
Proteobacteria;	0.16	13.53	4.38	26.58	26.58

Group 50
Average similarity: 52.17

Species	Av.Abund	Av.Sim	Sim/SD	Contrib%	Cum.%
Proteobacteria;	0.17	15.89	6.43	30.47	30.47

Group 20-25
Average similarity: 47.78

Species	Av.Abund	Av.Sim	Sim/SD	Contrib%	Cum.%
Proteobacteria;	0.17	14.03	4.17	29.37	29.37

Group 65-75
Average similarity: 55.11

Species	Av.Abund	Av.Sim	Sim/SD	Contrib%	Cum.%
Proteobacteria;	0.15	13.58	6.57	24.65	24.65

Group 100-110
Average similarity: 52.83

Species	Av.Abund	Av.Sim	Sim/SD	Contrib%	Cum.%
Proteobacteria;	0.15	12.92	4.58	24.46	24.46

Group 150
Average similarity: 53.39

Species	Av.Abund	Av.Sim	Sim/SD	Contrib%	Cum.%
Proteobacteria;	0.15	12.66	4.31	23.71	23.71

Group 200
Average similarity: 45.27

Species	Av.Abund	Av.Sim	Sim/SD	Contrib%	Cum.%
Proteobacteria;	0.18	15.06	5.17	33.27	33.27

Group 300
Average similarity: 42.43

Species	Av.Abund	Av.Sim	Sim/SD	Contrib%	Cum.%
Proteobacteria;	0.2	16.94	5.18	39.94	39.94

Group 500
Average similarity: 44.63

Species	Av.Abund	Av.Sim	Sim/SD	Contrib%	Cum.%
Proteobacteria;	0.19	16.26	6.08	36.44	36.44

Group 1000
Average similarity: 49.98

Species	Av.Abund	Av.Sim	Sim/SD	Contrib%	Cum.%
Proteobacteria;	0.22	20.07	8.68	40.16	40.16

Groups 0-3 & 50
Average dissimilarity = 49.10

	Group 0-3	Group 50				
Species	Av.Abund	Av.Abund	Av.Diss	Diss/SD	Contrib%	Cum.%
Proteobacteria; Gammaproteobacteria;	0.08	0.12	3.97	1.27	8.09	8.09
Planctomycetes; Phycisphaerae; MSBL9; SG8-4;	0.06	0.04	2.05	1.39	4.18	12.27
Proteobacteria;	0.16	0.17	1.92	1.35	3.91	16.18
Bacteroidetes; Bacteroidia; Bacteroidales; Bacteroidetes BD2-2;	0.02	0.02	0.77	1.32	1.56	17.74
Proteobacteria; Alphaproteobacteria; Rhodovibrionales; Kiloniellaceae;	0.01	0.02	0.73	1.24	1.48	19.22
Proteobacteria; Deltaproteobacteria; Desulfobacterales; Desulfobacteraceae; Sva0081 sediment group;	0.02	0.02	0.71	1.3	1.45	20.67

Groups 0-3 & 20-25
Average dissimilarity = 51.66

Species	Av.Abund	Av.Abund	Av.Diss	Diss/SD	Contrib%	Cum.%
Proteobacteria; Gammaproteobacteria;	0.08	0.09	3.52	1.09	6.8	6.8
Proteobacteria;	0.16	0.17	2.26	1.32	4.37	11.18
Planctomycetes; Phycisphaerae; MSBL9; SG8-4;	0.06	0.07	2.17	1.41	4.2	15.38
Proteobacteria; Alphaproteobacteria; Rhodovibrionales; Kiloniellaceae; uncultured;	0.02	0.02	0.86	1.12	1.67	17.05
Proteobacteria; Alphaproteobacteria; Rhodovibrionales; Kiloniellaceae;	0.01	0.02	0.81	0.97	1.56	18.61
Proteobacteria; Deltaproteobacteria; Desulfobacterales; Desulfobacteraceae; Sva0081 sediment group;	0.02	0.02	0.78	1.39	1.51	20.12

Groups 50 & 20-25
Average dissimilarity = 51.17

Species	Av.Abund	Av.Abund	Av.Diss	Diss/SD	Contrib%	Cum.%
Proteobacteria; Gammaproteobacteria;	0.12	0.09	4.45	1.21	8.7	8.7
Planctomycetes; Phycisphaerae; MSBL9; SG8-4;	0.04	0.07	2.13	1.46	4.16	12.86

Proteobacteria;	0.17	0.17	2.03	1.3	3.96	16.82
Proteobacteria; Deltaproteobacteria; Desulfobacterales; Desulfobacteraceae; Sva0081 sediment group;	0.02	0.02	0.81	1.39	1.58	18.4
Proteobacteria; Alphaproteobacteria; Rhodovibrionales; Kiloniellaceae; uncultured;	0.02	0.02	0.76	1.15	1.48	19.88
Bacteroidetes; Bacteroidia; Bacteroidales; Bacteroidetes BD2-2;	0.02	0.02	0.75	1.37	1.47	21.36

Groups 0-3 & 65-75
Average dissimilarity = 49.15

Species	Av.Abund	Av.Abund	Av.Diss	Diss/SD	Contrib%	Cum.%
Proteobacteria; Gammaproteobacteria;	0.08	0.08	2.79	1.12	5.67	5.67
Proteobacteria;	0.16	0.15	1.93	1.4	3.92	9.6
Planctomycetes; Phycisphaerae; MSBL9; SG8-4;	0.06	0.06	1.84	1.42	3.74	13.33
Actinobacteria; Coriobacteriia;	0.01	0.02	0.86	1.52	1.76	15.09
Proteobacteria; Alphaproteobacteria; Rhodovibrionales; Kiloniellaceae; uncultured;	0.02	0.02	0.79	1.18	1.61	16.7
Proteobacteria; Deltaproteobacteria; Desulfobacterales; Desulfobacteraceae; Sva0081 sediment group;	0.02	0.02	0.7	1.42	1.43	18.14
Firmicutes; Bacilli; Bacillales; Bacillaceae; Bacillus;	0	0.01	0.7	1.22	1.42	19.55
Bacteroidetes; Bacteroidia; Bacteroidales; Bacteroidetes BD2-2;	0.02	0.02	0.67	1.33	1.36	20.91

Groups 50 & 65-75
Average dissimilarity = 49.24

Species	Av.Abund	Av.Abund	Av.Diss	Diss/SD	Contrib%	Cum.%
Proteobacteria; Gammaproteobacteria;	0.12	0.08	3.88	1.16	7.89	7.89
Proteobacteria;	0.17	0.15	1.88	1.52	3.81	11.7
Planctomycetes; Phycisphaerae; MSBL9; SG8-4;	0.04	0.06	1.63	1.41	3.3	15
Actinobacteria; Coriobacteriia;	0.01	0.02	0.84	1.55	1.7	16.71
Proteobacteria; Deltaproteobacteria; Desulfobacterales; Desulfobacteraceae; Sva0081 sediment group;	0.02	0.02	0.74	1.49	1.51	18.22
Bacteroidetes; Bacteroidia; Bacteroidales; Bacteroidetes BD2-2;	0.02	0.02	0.71	1.45	1.43	19.65
Bacteroidetes; Bacteroidia; Cytophagales; Cyclobacteriaceae;	0.02	0.01	0.71	1.3	1.43	21.08

Groups 20-25 & 65-75
Average dissimilarity = 48.98

Species	Av.Abund	Av.Abund	Av.Diss	Diss/SD	Contrib%	Cum.%
Proteobacteria; Gammaproteobacteria;	0.09	0.08	3.32	0.96	6.77	6.77
Proteobacteria;	0.17	0.15	2.17	1.35	4.43	11.21
Planctomycetes; Phycisphaerae; MSBL9; SG8-4;	0.07	0.06	1.8	1.42	3.67	14.87
Proteobacteria; Alphaproteobacteria; Rhodovibrionales; Kiloniellaceae; uncultured;	0.02	0.02	0.88	1.16	1.79	16.67
Actinobacteria; Coriobacteriia;	0.02	0.02	0.78	1.44	1.6	18.27
Proteobacteria; Deltaproteobacteria; Desulfobacterales; Desulfobacteraceae; Sva0081 sediment group;	0.02	0.02	0.75	1.26	1.53	19.8
Firmicutes; Bacilli; Bacillales; Bacillaceae; Bacillus;	0.01	0.01	0.72	1.31	1.46	21.26

Groups 0-3 & 100-110
Average dissimilarity = 49.46

Species	Av.Abund	Av.Abund	Av.Diss	Diss/SD	Contrib%	Cum.%
Proteobacteria; Gammaproteobacteria;	0.08	0.06	2.63	1.13	5.31	5.31
Planctomycetes; Phycisphaerae; MSBL9; SG8-4;	0.06	0.08	2.09	1.32	4.22	9.52
Proteobacteria;	0.16	0.15	2.03	1.41	4.1	13.62
Proteobacteria; Alphaproteobacteria; Rhodovibrionales; Kiloniellaceae;	0.01	0.02	0.81	0.83	1.63	15.26
Proteobacteria; Alphaproteobacteria; Rhodobacterales; Rhodobacteraceae;	0.01	0.02	0.79	0.59	1.6	16.86
Bacteroidetes; Bacteroidia; Bacteroidales; Bacteroidetes BD2-2;	0.02	0.02	0.78	1.35	1.58	18.44
Proteobacteria; Alphaproteobacteria; Rhodovibrionales; Kiloniellaceae; uncultured;	0.02	0.02	0.74	1.21	1.49	19.93
Bacteroidetes; Bacteroidia; Cytophagales; Cyclobacteriaceae;	0.01	0.01	0.62	1.07	1.25	21.18

Groups 50 & 100-110
Average dissimilarity = 50.64

Species	Av.Abund	Av.Abund	Av.Diss	Diss/SD	Contrib%	Cum.%
Proteobacteria; Gammaproteobacteria;	0.12	0.06	4.06	1.17	8.02	8.02
Planctomycetes; Phycisphaerae; MSBL9; SG8-4;	0.04	0.08	2.13	1.28	4.2	12.21
Proteobacteria;	0.17	0.15	1.98	1.35	3.91	16.12
Bacteroidetes; Bacteroidia; Bacteroidales; Bacteroidetes BD2-2;	0.02	0.02	0.8	1.4	1.58	17.71
Proteobacteria; Alphaproteobacteria; Rhodobacterales; Rhodobacteraceae;	0.01	0.02	0.74	0.55	1.46	19.17
Proteobacteria; Alphaproteobacteria; Rhodovibrionales; Kiloniellaceae;	0.02	0.02	0.74	0.83	1.45	20.62

Groups 20-25 & 100-110
Average dissimilarity = 49.71

Species	Av.Abund	Av.Abund	Av.Diss	Diss/SD	Contrib%	Cum.%
Proteobacteria; Gammaproteobacteria;	0.09	0.06	3.1	0.89	6.24	6.24
Proteobacteria;	0.17	0.15	2.26	1.32	4.55	10.79
Planctomycetes; Phycisphaerae; MSBL9; SG8-4;	0.07	0.08	1.91	1.24	3.85	14.63
Proteobacteria; Alphaproteobacteria; Rhodovibrionales; Kiloniellaceae; uncultured;	0.02	0.02	0.81	1.13	1.64	16.27
Proteobacteria; Alphaproteobacteria; Rhodovibrionales; Kiloniellaceae;	0.02	0.02	0.79	0.74	1.59	17.86
Proteobacteria; Alphaproteobacteria; Rhodobacterales; Rhodobacteraceae;	0.01	0.02	0.78	0.57	1.58	19.44
Bacteroidetes; Bacteroidia; Bacteroidales; Bacteroidetes BD2-2;	0.02	0.02	0.76	1.37	1.53	20.97

Groups 65-75 & 100-110
Average dissimilarity = 46.22

Species	Av.Abund	Av.Abund	Av.Diss	Diss/SD	Contrib%	Cum.%
Proteobacteria; Gammaproteobacteria;	0.08	0.06	2.38	0.94	5.16	5.16
Proteobacteria;	0.15	0.15	1.89	1.44	4.09	9.24
Planctomycetes; Phycisphaerae; MSBL9; SG8-4;	0.06	0.08	1.62	1.13	3.5	12.75
Proteobacteria; Alphaproteobacteria; Rhodovibrionales; Kiloniellaceae; uncultured;	0.02	0.02	0.76	1.26	1.63	14.38
Actinobacteria; Coriobacteriia;	0.02	0.02	0.72	1.58	1.56	15.94
Proteobacteria; Alphaproteobacteria; Rhodobacterales; Rhodobacteraceae;	0.01	0.02	0.72	0.51	1.55	17.49
Firmicutes; Bacilli; Bacillales; Bacillaceae; Bacillus;	0.01	0.01	0.71	1.29	1.53	19.02
Bacteroidetes; Bacteroidia; Bacteroidales; Bacteroidetes BD2-2;	0.02	0.02	0.69	1.32	1.5	20.51

Groups 0-3 & 150
Average dissimilarity = 50.77

Species	Av.Abund	Av.Abund	Av.Diss	Diss/SD	Contrib%	Cum.%
Proteobacteria; Gammaproteobacteria;	0.08	0.09	3.16	1.1	6.23	6.23
Proteobacteria;	0.16	0.15	2.11	1.41	4.15	10.38
Planctomycetes; Phycisphaerae; MSBL9; SG8-4;	0.06	0.05	1.85	1.41	3.65	14.03
Proteobacteria; Alphaproteobacteria; Rhodovibrionales; Kiloniellaceae; uncultured;	0.02	0.02	0.81	1.27	1.6	15.62
Actinobacteria; Coriobacteriia;	0.01	0.02	0.8	1.74	1.58	17.21
Proteobacteria; Alphaproteobacteria; Rhodovibrionales; Kiloniellaceae;	0.01	0.02	0.7	1.19	1.39	18.59
Chlamydiae; Chlamydiae; Chlamydiales;	0.01	0.02	0.67	1.24	1.32	19.91
Dependentiae; Babeliae; Babeliales; Vermiphilaceae;	0.01	0.02	0.64	1.11	1.27	21.17

Groups 50 & 150
Average dissimilarity = 50.30

Species	Av.Abund	Av.Abund	Av.Diss	Diss/SD	Contrib%	Cum.%
Proteobacteria; Gammaproteobacteria;	0.12	0.09	4.14	1.19	8.23	8.23
Proteobacteria;	0.17	0.15	2.05	1.34	4.07	12.3
Planctomycetes; Phycisphaerae; MSBL9; SG8-4;	0.04	0.05	1.51	1.44	3	15.3
Actinobacteria; Coriobacteriia;	0.01	0.02	0.76	1.7	1.52	16.82
Chlamydiae; Chlamydiae; Chlamydiales;	0.01	0.02	0.72	1.26	1.43	18.26
Proteobacteria; Alphaproteobacteria; Rhodovibrionales; Kiloniellaceae; uncultured;	0.02	0.02	0.72	1.49	1.43	19.68
Bacteroidetes; Bacteroidia; Cytophagales; Cyclobacteriaceae;	0.02	0.01	0.68	1.27	1.34	21.03

Groups 20-25 & 150
Average dissimilarity = 49.81

Species	Av.Abund	Av.Abund	Av.Diss	Diss/SD	Contrib%	Cum.%
Proteobacteria; Gammaproteobacteria;	0.09	0.09	3.62	0.97	7.27	7.27
Proteobacteria;	0.17	0.15	2.34	1.33	4.69	11.96
Planctomycetes; Phycisphaerae; MSBL9; SG8-4;	0.07	0.05	1.89	1.45	3.79	15.75
Proteobacteria; Alphaproteobacteria; Rhodovibrionales; Kiloniellaceae; uncultured;	0.02	0.02	0.88	1.18	1.77	17.51
Dependentiae; Babeliae; Babeliales; Vermiphilaceae;	0.01	0.02	0.75	1.15	1.5	19.01
Proteobacteria; Deltaproteobacteria; Desulfobacterales; Desulfobacteraceae; Sva0081 sediment group;	0.02	0.02	0.7	1.39	1.41	20.42

Groups 65-75 & 150
Average dissimilarity = 45.08

Species	Av.Abund	Av.Abund	Av.Diss	Diss/SD	Contrib%	Cum.%
Proteobacteria; Gammaproteobacteria;	0.08	0.09	2.93	0.93	6.51	6.51
Proteobacteria;	0.15	0.15	1.98	1.45	4.38	10.9
Planctomycetes; Phycisphaerae; MSBL9; SG8-4;	0.06	0.05	1.31	1.26	2.91	13.8
Proteobacteria; Alphaproteobacteria; Rhodovibrionales; Kiloniellaceae; uncultured;	0.02	0.02	0.83	1.31	1.83	15.64
Actinobacteria; Coriobacteriia;	0.02	0.02	0.69	1.3	1.54	17.18
Firmicutes; Bacilli; Bacillales; Bacillaceae; Bacillus;	0.01	0.01	0.68	1.35	1.52	18.69
Chlamydiae; Chlamydiae; Chlamydiales;	0.02	0.02	0.68	1.35	1.52	20.21

Groups 100-110 & 150
Average dissimilarity = 47.19

Species	Av.Abund	Av.Abund	Av.Diss	Diss/SD	Contrib%	Cum.%
Proteobacteria; Gammaproteobacteria;	0.06	0.09	2.76	0.89	5.86	5.86
Proteobacteria;	0.15	0.15	2.01	1.38	4.25	10.11
Planctomycetes; Phycisphaerae; MSBL9; SG8-4;	0.08	0.05	1.79	1.17	3.79	13.9
Proteobacteria; Alphaproteobacteria; Rhodovibrionales; Kiloniellaceae; uncultured;	0.02	0.02	0.74	1.33	1.57	15.48
Proteobacteria; Alphaproteobacteria; Rhodobacterales; Rhodobacteraceae;	0.02	0.01	0.74	0.54	1.57	17.05
Dependentiae; Babeliae; Babeliales; Vermiphilaceae;	0.01	0.02	0.74	1.2	1.56	18.61
Proteobacteria; Alphaproteobacteria; Rhodovibrionales; Kiloniellaceae;	0.02	0.02	0.7	0.77	1.48	20.08

Groups 0-3 & 200
Average dissimilarity = 51.84

Species	Av.Abund	Av.Abund	Av.Diss	Diss/SD	Contrib%	Cum.%
Proteobacteria; Gammaproteobacteria;	0.08	0.09	4.07	1.05	7.85	7.85
Proteobacteria;	0.16	0.18	2.31	1.35	4.45	12.31
Planctomycetes; Phycisphaerae; MSBL9; SG8-4;	0.06	0.06	2.12	1.34	4.1	16.4
Planctomycetes; Phycisphaerae; MSBL9; L21-RPul-D3;	0.01	0.02	0.92	1.38	1.77	18.18
Proteobacteria; Deltaproteobacteria; Desulfobacterales; Desulfobacteraceae; Sva0081 sediment group;	0.02	0.02	0.82	1.25	1.59	19.76
Proteobacteria; Alphaproteobacteria; Rhodovibrionales; Kiloniellaceae;	0.01	0.02	0.78	1.12	1.5	21.27

Groups 50 & 200
Average dissimilarity = 51.59

Species	Av.Abund	Av.Abund	Av.Diss	Diss/SD	Contrib%	Cum.%
Proteobacteria; Gammaproteobacteria;	0.12	0.09	5.05	1.23	9.78	9.78
Planctomycetes; Phycisphaerae; MSBL9; SG8-4;	0.04	0.06	1.96	1.34	3.79	13.58
Proteobacteria;	0.17	0.18	1.96	1.37	3.79	17.37
Planctomycetes; Phycisphaerae; MSBL9; L21-RPul-D3;	0.01	0.02	0.97	1.4	1.89	19.25
Proteobacteria; Deltaproteobacteria; Desulfobacterales; Desulfobacteraceae; Sva0081 sediment group;	0.02	0.02	0.86	1.28	1.67	20.92

Groups 20-25 & 200
Average dissimilarity = 53.27

Species	Av.Abund	Av.Abund	Av.Diss	Diss/SD	Contrib%	Cum.%
Proteobacteria; Gammaproteobacteria;	0.09	0.09	4.35	0.94	8.17	8.17
Proteobacteria;	0.17	0.18	2.4	1.32	4.5	12.67
Planctomycetes; Phycisphaerae; MSBL9; SG8-4;	0.07	0.06	2.1	1.36	3.94	16.61
Planctomycetes; Phycisphaerae; MSBL9; L21-RPul-D3;	0.01	0.02	0.94	1.44	1.77	18.38
Proteobacteria; Deltaproteobacteria; Desulfobacterales; Desulfobacteraceae; Sva0081 sediment group;	0.02	0.02	0.89	1.35	1.67	20.05

Groups 65-75 & 200
Average dissimilarity = 51.77

Species	Av.Abund	Av.Abund	Av.Diss	Diss/SD	Contrib%	Cum.%
Proteobacteria; Gammaproteobacteria;	0.08	0.09	3.94	0.97	7.61	7.61
Proteobacteria;	0.15	0.18	2.22	1.34	4.28	11.89
Planctomycetes; Phycisphaerae; MSBL9; SG8-4;	0.06	0.06	1.69	1.23	3.26	15.15
Planctomycetes; Phycisphaerae; MSBL9; L21-RPul-D3;	0.01	0.02	0.89	1.46	1.73	16.87
Proteobacteria; Deltaproteobacteria; Desulfobacterales; Desulfobacteraceae; Sva0081 sediment group;	0.02	0.02	0.84	1.33	1.62	18.49
Actinobacteria; Coriobacteriia;	0.02	0.01	0.82	1.47	1.59	20.08

Groups 100-110 & 200
Average dissimilarity = 51.82

Species	Av.Abund	Av.Abund	Av.Diss	Diss/SD	Contrib%	Cum.%
Proteobacteria; Gammaproteobacteria;	0.06	0.09	3.53	0.83	6.81	6.81
Proteobacteria;	0.15	0.18	2.35	1.32	4.53	11.34
Planctomycetes; Phycisphaerae; MSBL9; SG8-4;	0.08	0.06	1.98	1.2	3.82	15.17
Planctomycetes; Phycisphaerae; MSBL9; L21-RPul-D3;	0.01	0.02	0.87	1.5	1.68	16.84
Bacteroidetes; Bacteroidia; Bacteroidales; Bacteroidetes BD2-2;	0.02	0.01	0.86	1.39	1.65	18.5
Proteobacteria; Alphaproteobacteria; Rhodobacterales; Rhodobacteraceae;	0.02	0.01	0.83	0.62	1.6	20.09

Groups 150 & 200
Average dissimilarity = 53.05

Species	Av.Abund	Av.Abund	Av.Diss	Diss/SD	Contrib%	Cum.%
Proteobacteria; Gammaproteobacteria;	0.09	0.09	4.17	0.97	7.86	7.86
Proteobacteria;	0.15	0.18	2.45	1.37	4.62	12.48
Planctomycetes; Phycisphaerae; MSBL9; SG8-4;	0.05	0.06	1.7	1.24	3.21	15.69
Planctomycetes; Phycisphaerae; MSBL9; L21-RPul-D3;	0.01	0.02	0.87	1.46	1.63	17.32
Proteobacteria; Alphaproteobacteria; Rhodovibrionales; Kiloniellaceae; uncultured;	0.02	0.02	0.77	1.59	1.45	18.77
Proteobacteria; Deltaproteobacteria; Desulfobacterales; Desulfobacteraceae; Sva0081 sediment group;	0.02	0.02	0.76	1.26	1.43	20.19

Groups 0-3 & 300
Average dissimilarity = 56.26

Species	Av.Abund	Av.Abund	Av.Diss	Diss/SD	Contrib%	Cum.%
Proteobacteria; Gammaproteobacteria;	0.08	0.09	4.51	1.18	8.01	8.01
Proteobacteria;	0.16	0.2	2.7	1.37	4.8	12.81
Planctomycetes; Phycisphaerae; MSBL9; SG8-4;	0.06	0.07	2.54	1.41	4.51	17.32
Planctomycetes; Phycisphaerae; MSBL9; L21-RPul-D3;	0.01	0.04	2.09	1.18	3.71	21.03

Groups 50 & 300
Average dissimilarity = 56.27

Species	Av.Abund	Av.Abund	Av.Diss	Diss/SD	Contrib%	Cum.%
Proteobacteria; Gammaproteobacteria;	0.12	0.09	5.41	1.35	9.61	9.61
Planctomycetes; Phycisphaerae; MSBL9; SG8-4;	0.04	0.07	2.45	1.38	4.36	13.97
Planctomycetes; Phycisphaerae; MSBL9; L21-RPul-D3;	0.01	0.04	2.11	1.12	3.75	17.72
Proteobacteria;	0.17	0.2	2.1	1.29	3.73	21.45

Groups 20-25 & 300
Average dissimilarity = 57.01

Species	Av.Abund	Av.Abund	Av.Diss	Diss/SD	Contrib%	Cum.%
Proteobacteria; Gammaproteobacteria;	0.09	0.09	4.78	1.05	8.38	8.38
Proteobacteria;	0.17	0.2	2.68	1.36	4.7	13.08
Planctomycetes; Phycisphaerae; MSBL9; SG8-4;	0.07	0.07	2.49	1.42	4.37	17.44
Planctomycetes; Phycisphaerae; MSBL9; L21-RPul-D3;	0.01	0.04	2.09	1.17	3.67	21.11

Groups 65-75 & 300

Average dissimilarity = 56.32

Species	Av.Abund	Av.Abund	Av.Diss	Diss/SD	Contrib%	Cum.%
Proteobacteria; Gammaproteobacteria;	0.08	0.09	4.37	1.09	7.77	7.77
Proteobacteria;	0.15	0.2	2.7	1.46	4.8	12.57
Planctomycetes; Phycisphaerae; MSBL9; SG8-4;	0.06	0.07	2.24	1.43	3.97	16.54
Planctomycetes; Phycisphaerae; MSBL9; L21-RPul-D3;	0.01	0.04	2.09	1.2	3.71	20.25

Groups 100-110 & 300
Average dissimilarity = 56.61

Species	Av.Abund	Av.Abund	Av.Diss	Diss/SD	Contrib%	Cum.%
Proteobacteria; Gammaproteobacteria;	0.06	0.09	4.03	0.95	7.11	7.11
Proteobacteria;	0.15	0.2	2.85	1.39	5.04	12.15
Planctomycetes; Phycisphaerae; MSBL9; SG8-4;	0.08	0.07	2.41	1.42	4.26	16.42
Planctomycetes; Phycisphaerae; MSBL9; L21-RPul-D3;	0.01	0.04	2.08	1.24	3.67	20.09

Groups 150 & 300
Average dissimilarity = 58.06

Species	Av.Abund	Av.Abund	Av.Diss	Diss/SD	Contrib%	Cum.%
Proteobacteria; Gammaproteobacteria;	0.09	0.09	4.56	1.06	7.86	7.86
Proteobacteria;	0.15	0.2	2.9	1.38	4.99	12.86
Planctomycetes; Phycisphaerae; MSBL9; SG8-4;	0.05	0.07	2.28	1.4	3.92	16.78
Planctomycetes; Phycisphaerae; MSBL9; L21-RPul-D3;	0.01	0.04	2.07	1.26	3.56	20.34

Groups 200 & 300
Average dissimilarity = 55.99

Species	Av.Abund	Av.Abund	Av.Diss	Diss/SD	Contrib%	Cum.%
Proteobacteria; Gammaproteobacteria;	0.09	0.09	5.04	0.96	9	9
Proteobacteria;	0.18	0.2	2.48	1.39	4.43	13.43
Planctomycetes; Phycisphaerae; MSBL9; SG8-4;	0.06	0.07	2.48	1.37	4.43	17.86
Planctomycetes; Phycisphaerae; MSBL9; L21-RPul-D3;	0.02	0.04	2.02	1.38	3.61	21.47

Groups 0-3 & 500
Average dissimilarity = 56.27

Species	Av.Abund	Av.Abund	Av.Diss	Diss/SD	Contrib%	Cum.%
Proteobacteria; Gammaproteobacteria;	0.08	0.08	3.78	1.13	6.71	6.71
Proteobacteria;	0.16	0.19	2.54	1.31	4.52	11.23
Planctomycetes; Phycisphaerae; MSBL9; L21-RPul-D3;	0.01	0.06	2.38	1.14	4.24	15.46
Planctomycetes; Phycisphaerae; MSBL9; SG8-4;	0.06	0.07	2.3	1.38	4.09	19.55
Proteobacteria; Deltaproteobacteria; Desulfobacterales; Desulfobacteraceae; Sva0081 sediment group;	0.02	0.04	1.68	1.85	2.99	22.54

Groups 50 & 500
Average dissimilarity = 57.07

Species	Av.Abund	Av.Abund	Av.Diss	Diss/SD	Contrib%	Cum.%
Proteobacteria; Gammaproteobacteria;	0.12	0.08	4.86	1.28	8.52	8.52
Planctomycetes; Phycisphaerae; MSBL9; L21-RPul-D3;	0.01	0.06	2.52	1.2	4.42	12.94
Planctomycetes; Phycisphaerae; MSBL9; SG8-4;	0.04	0.07	2.31	1.48	4.06	17
Proteobacteria;	0.17	0.19	2.08	1.36	3.65	20.65

Groups 20-25 & 500
Average dissimilarity = 56.66

Species	Av.Abund	Av.Abund	Av.Diss	Diss/SD	Contrib%	Cum.%
Proteobacteria; Gammaproteobacteria;	0.09	0.08	4.11	0.98	7.26	7.26
Proteobacteria;	0.17	0.19	2.57	1.32	4.54	11.79
Planctomycetes; Phycisphaerae; MSBL9; L21-RPul-D3;	0.01	0.06	2.42	1.16	4.27	16.06
Planctomycetes; Phycisphaerae; MSBL9; SG8-4;	0.07	0.07	2.17	1.29	3.83	19.89
Proteobacteria; Deltaproteobacteria; Desulfobacterales; Desulfobacteraceae; Sva0081 sediment group;	0.02	0.04	1.65	1.79	2.91	22.8

Groups 65-75 & 500
Average dissimilarity = 55.84

Species	Av.Abund	Av.Abund	Av.Diss	Diss/SD	Contrib%	Cum.%
Proteobacteria; Gammaproteobacteria;	0.08	0.08	3.65	1.04	6.54	6.54
Proteobacteria;	0.15	0.19	2.49	1.32	4.45	11
Planctomycetes; Phycisphaerae; MSBL9; L21-RPul-D3;	0.01	0.06	2.38	1.15	4.27	15.26
Planctomycetes; Phycisphaerae; MSBL9; SG8-4;	0.06	0.07	1.96	1.39	3.51	18.77
Proteobacteria; Deltaproteobacteria; Desulfobacterales; Desulfobacteraceae; Sva0081 sediment group;	0.02	0.04	1.68	1.88	3	21.78

Groups 100-110 & 500
Average dissimilarity = 55.43

Species	Av.Abund	Av.Abund	Av.Diss	Diss/SD	Contrib%	Cum.%
Proteobacteria; Gammaproteobacteria;	0.06	0.08	3.23	0.9	5.83	5.83
Proteobacteria;	0.15	0.19	2.66	1.32	4.8	10.64
Planctomycetes; Phycisphaerae; MSBL9; L21-RPul-D3;	0.01	0.06	2.31	1.13	4.17	14.81

Planctomycetes; Phycisphaerae; MSBL9; SG8-4;	0.08	0.07	2.03	1.23	3.65	18.46
Proteobacteria; Deltaproteobacteria; Desulfobacterales; Desulfobacteraceae; Sva0081 sediment group;	0.02	0.04	1.62	2.42	2.93	21.39

Groups 150 & 500
Average dissimilarity = 57.39

Species	Av.Abund	Av.Abund	Av.Diss	Diss/SD	Contrib%	Cum.%
Proteobacteria; Gammaproteobacteria;	0.09	0.08	3.91	1.02	6.81	6.81
Proteobacteria;	0.15	0.19	2.74	1.35	4.78	11.59
Planctomycetes; Phycisphaerae; MSBL9; L21-RPul-D3;	0.01	0.06	2.27	1.11	3.96	15.55
Planctomycetes; Phycisphaerae; MSBL9; SG8-4;	0.05	0.07	2.07	1.45	3.61	19.16
Proteobacteria; Deltaproteobacteria; Desulfobacterales; Desulfobacteraceae; Sva0081 sediment group;	0.02	0.04	1.69	2.06	2.94	22.09

Groups 200 & 500
Average dissimilarity = 55.16

Species	Av.Abund	Av.Abund	Av.Diss	Diss/SD	Contrib%	Cum.%
Proteobacteria; Gammaproteobacteria;	0.09	0.08	4.37	0.89	7.92	7.92
Proteobacteria;	0.18	0.19	2.39	1.31	4.32	12.25
Planctomycetes; Phycisphaerae; MSBL9; SG8-4;	0.06	0.07	2.21	1.3	4.01	16.26
Planctomycetes; Phycisphaerae; MSBL9; L21-RPul-D3;	0.02	0.06	2.02	1.02	3.66	19.92
Proteobacteria; Deltaproteobacteria; Desulfobacterales; Desulfobacteraceae; Sva0081 sediment group;	0.02	0.04	1.59	1.73	2.88	22.81

Groups 300 & 500
Average dissimilarity = 54.95

Species	Av.Abund	Av.Abund	Av.Diss	Diss/SD	Contrib%	Cum.%
Proteobacteria; Gammaproteobacteria;	0.09	0.08	4.78	0.97	8.69	8.69
Planctomycetes; Phycisphaerae; MSBL9; SG8-4;	0.07	0.07	2.59	1.4	4.72	13.41
Planctomycetes; Phycisphaerae; MSBL9; L21-RPul-D3;	0.04	0.06	2.52	1.37	4.58	17.99
Proteobacteria;	0.2	0.19	2.41	1.41	4.38	22.38

Groups 0-3 & 1000
Average dissimilarity = 55.28

Species	Av.Abund	Av.Abund	Av.Diss	Diss/SD	Contrib%	Cum.%
Proteobacteria; Gammaproteobacteria;	0.08	0.17	5.93	1.37	10.73	10.73
Proteobacteria;	0.16	0.22	3.16	1.59	5.72	16.45
Planctomycetes; Phycisphaerae; MSBL9; SG8-4;	0.06	0.06	2.47	1.39	4.47	20.92

Groups 50 & 1000
Average dissimilarity = 52.50

Species	Av.Abund	Av.Abund	Av.Diss	Diss/SD	Contrib%	Cum.%
Proteobacteria; Gammaproteobacteria;	0.12	0.17	5.73	1.43	10.92	10.92
Proteobacteria;	0.17	0.22	2.35	1.51	4.48	15.39
Planctomycetes; Phycisphaerae; MSBL9; SG8-4;	0.04	0.06	2.26	1.26	4.31	19.7
Proteobacteria; Deltaproteobacteria; Desulfobacterales; Desulfobacteraceae; Sva0081 sediment group;	0.02	0.03	1.43	1.32	2.72	22.43

Groups 20-25 & 1000
Average dissimilarity = 55.87

Species	Av.Abund	Av.Abund	Av.Diss	Diss/SD	Contrib%	Cum.%
Proteobacteria; Gammaproteobacteria;	0.09	0.17	6.26	1.35	11.2	11.2
Proteobacteria;	0.17	0.22	3.02	1.54	5.41	16.61
Planctomycetes; Phycisphaerae; MSBL9; SG8-4;	0.07	0.06	2.51	1.52	4.48	21.09

Groups 65-75 & 1000
Average dissimilarity = 55.66

Species	Av.Abund	Av.Abund	Av.Diss	Diss/SD	Contrib%	Cum.%
Proteobacteria; Gammaproteobacteria;	0.08	0.17	6.02	1.37	10.81	10.81
Proteobacteria;	0.15	0.22	3.24	1.79	5.82	16.63
Planctomycetes; Phycisphaerae; MSBL9; SG8-4;	0.06	0.06	2.17	1.44	3.9	20.53

Groups 100-110 & 1000
Average dissimilarity = 57.09

Species	Av.Abund	Av.Abund	Av.Diss	Diss/SD	Contrib%	Cum.%
Proteobacteria; Gammaproteobacteria;	0.06	0.17	6.35	1.35	11.12	11.12
Proteobacteria;	0.15	0.22	3.46	1.74	6.06	17.18
Planctomycetes; Phycisphaerae; MSBL9; SG8-4;	0.08	0.06	2.47	1.52	4.33	21.51

Groups 150 & 1000
Average dissimilarity = 57.20

Species	Av.Abund	Av.Abund	Av.Diss	Diss/SD	Contrib%	Cum.%
Proteobacteria; Gammaproteobacteria;	0.09	0.17	6.04	1.35	10.56	10.56
Proteobacteria;	0.15	0.22	3.48	1.68	6.08	16.64
Planctomycetes; Phycisphaerae; MSBL9; SG8-4;	0.05	0.06	2.13	1.32	3.72	20.36

Groups 200 & 1000
Average dissimilarity = 55.05

Species	Av.Abund	Av.Abund	Av.Diss	Diss/SD	Contrib%	Cum.%
Proteobacteria; Gammaproteobacteria;	0.09	0.17	6.68	1.33	12.13	12.13
Proteobacteria;	0.18	0.22	2.64	1.51	4.8	16.93
Planctomycetes; Phycisphaerae; MSBL9; SG8-4;	0.06	0.06	2.43	1.39	4.41	21.34

Groups 300 & 1000
Average dissimilarity = 54.72

Species	Av.Abund	Av.Abund	Av.Diss	Diss/SD	Contrib%	Cum.%
Proteobacteria; Gammaproteobacteria;	0.09	0.17	6.89	1.39	12.59	12.59
Planctomycetes; Phycisphaerae; MSBL9; SG8-4;	0.07	0.06	2.76	1.38	5.04	17.63
Proteobacteria;	0.2	0.22	2.16	1.4	3.95	21.58

Groups 500 & 1000
Average dissimilarity = 55.60

Species	Av.Abund	Av.Abund	Av.Diss	Diss/SD	Contrib%	Cum.%
Proteobacteria; Gammaproteobacteria;	0.08	0.17	6.62	1.35	11.9	11.9
Planctomycetes; Phycisphaerae; MSBL9; SG8-4;	0.07	0.06	2.65	1.54	4.77	16.67
Proteobacteria;	0.19	0.22	2.4	1.59	4.31	20.98

Table S4. Model summary for machine learning analysis predicting proximity to metal-hulled shipwrecks *Alcoa Puritan* and *Halo* .

Sample ID	Site	Prediction	Actual
D-188	Alcoa Puritan	42.846	100
D-195	Alcoa Puritan	47.171	25
D-194	Alcoa Puritan	60.746	25
D-205	Alcoa Puritan	60.416	100
D-208	Alcoa Puritan	26.652	100
D-189	Alcoa Puritan	64.692	2.5
D-191	Alcoa Puritan	57.794	2.5
D-157	Alcoa Puritan	27.873	50
D-167	Alcoa Puritan	29.233	6
D-170	Alcoa Puritan	30.321	3
D-165	Alcoa Puritan	54.981	6
Halo-500	Halo	84.91	100
Halo-4	Halo	25.32	2
Halo-168	Halo	16.89	2
Halo-286	Halo	51.888	150
Halo-176	Halo	84.41	200
Halo-495	Halo	68.28	100
Halo-1	Halo	20.798	2
Halo-181	Halo	44.248	200
Halo-488	Halo	67.14	50
Halo-498	Halo	65.578	100
Halo-499	Halo	105.91	100
Halo-167	Halo	9.39	2

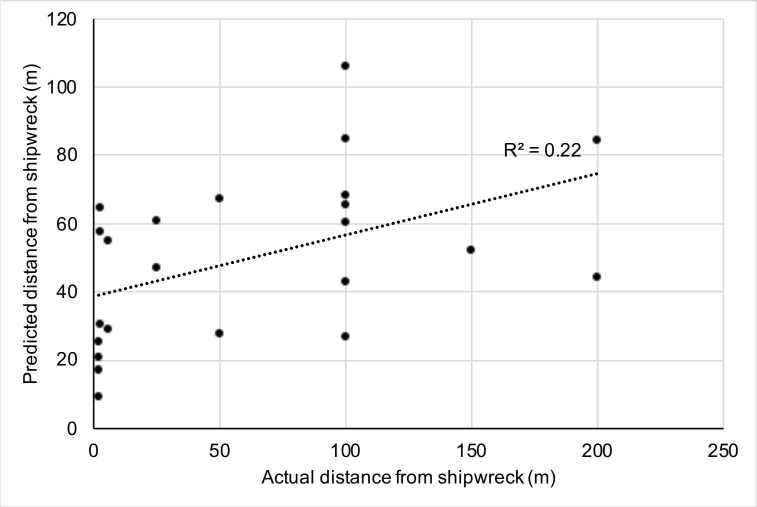

SUMMARY OUTPUT

Regression Statistics	
Multiple R	0.47
R Square	0.22
Adjusted R Squ	0.19
Standard Error	58
Observations	23

ANOVA

	df	*SS*	*MS*	*F*	*Significance F*
Regression	1	20127.08	20127.08	6.03	0.02
Residual	21	70072.24	3336.77		
Total	22	90199.33			

	Coefficients	*Standard Error*	*t Stat*	*P-value*	*Lower 95%*	*Upper 95%*	*Lower 95.0%*	*Upper 95.0%*
Intercept	0.20	27.93	0.01	0.99	-57.88	58.28	-57.88	58.28
X Variable 1	1.24	0.51	2.46	0.02	0.19	2.29	0.19	2.29
