# Supplementary figures and images for: Deep-sea shipwrecks represent island-like ecosystems for marine microbiomes
Source: ISME J. 2021 Apr 22;15(10):2883–91. doi: 10.1038/s41396-021-00978-y (PMC8443566; doi:10.1038/s41396-021-00978-y)

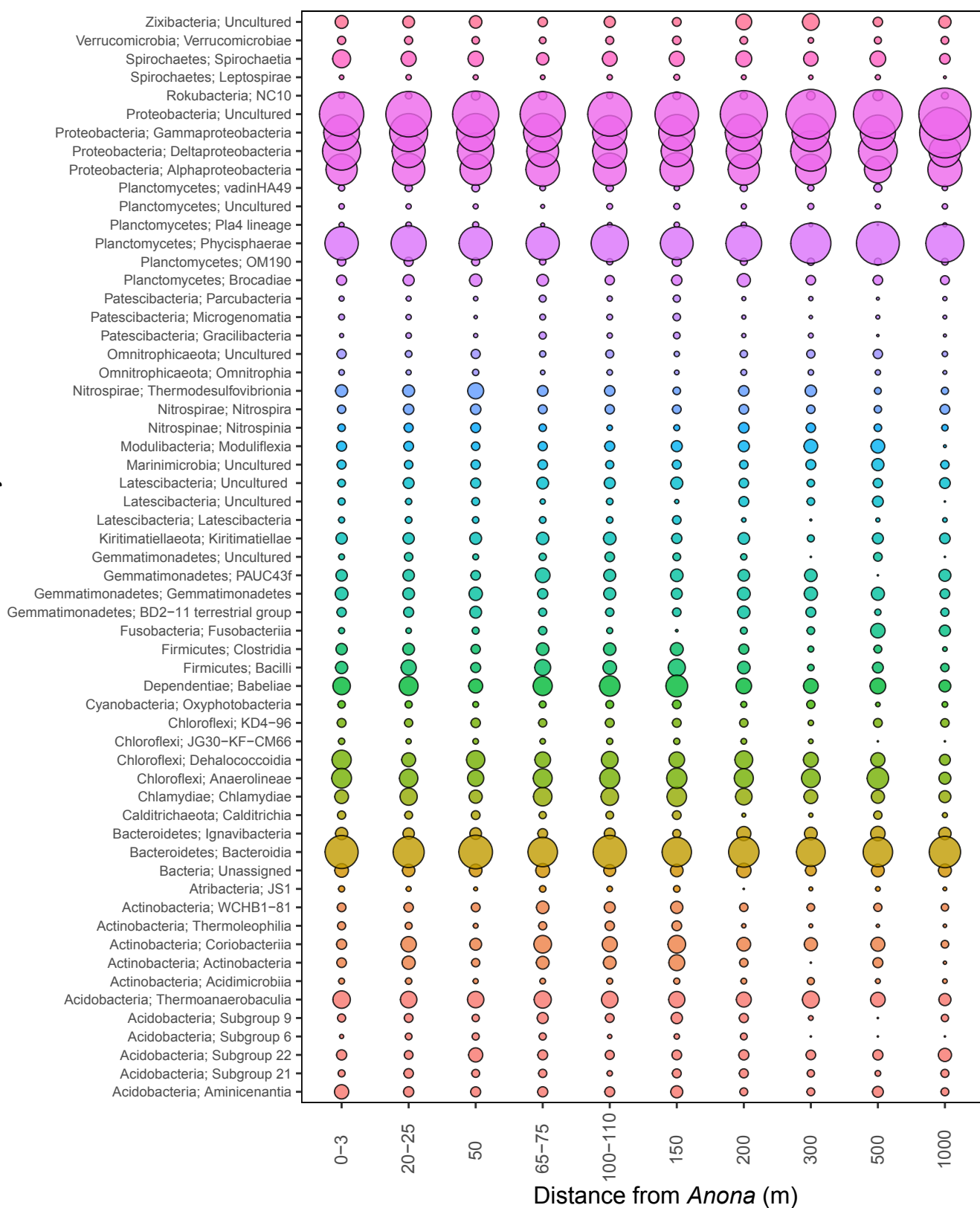

Relative Abundance

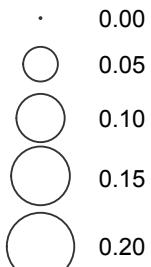

Phylum

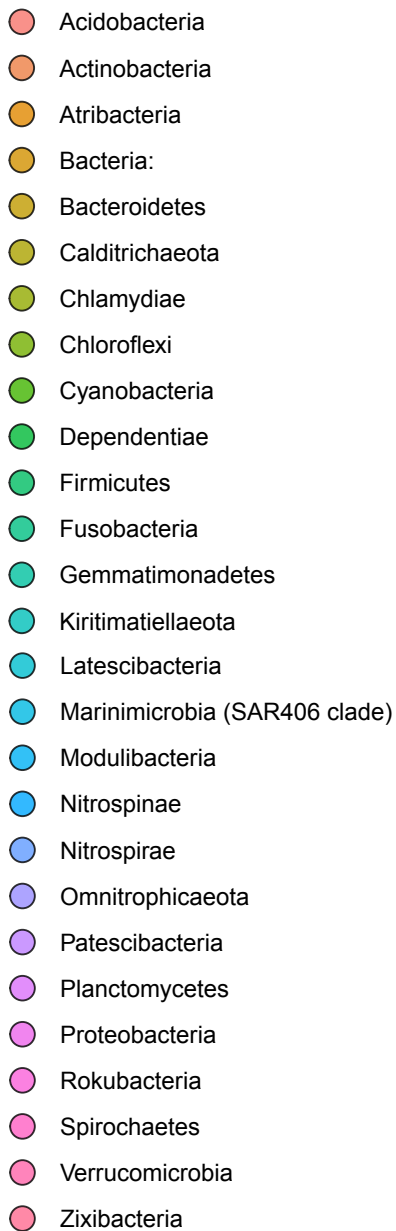

Supplement: Supplementary file 2 — Figure S1 [file 41396_2021_978_MOESM2_ESM.pdf]
